# Financial and economic uncertainties and their effects on the economy

**DOI:** 10.1007/s10663-023-09570-3

**Published:** 2023-03-20

**Authors:** Ines Fortin, Jaroslava Hlouskova, Leopold Sögner

**Affiliations:** 1 Macroeconomics and Business Cycles, Institute for Advanced Studies, Vienna, Austria; 2 Department of Economics, Faculty of National Economy, University of Economics, Bratislava, Slovakia; 3 Vienna Graduate School of Finance (VGSF), Vienna, Austria

**Keywords:** Financial uncertainty, Economic uncertainty, Financial crisis, Forecasting, C53, G01, G20, E44

## Abstract

We estimate new indices measuring financial and economic uncertainty in the euro area, Germany, France, the United Kingdom and Austria, following the approach of Jurado et al. (Am Econ Rev 105:1177–1216, 2015), which measures uncertainty by the degree of predictability. We perform an impulse response analysis in a vector error correction framework, where we focus on the impact of both local and global uncertainty shocks on industrial production, employment and the stock market. We find that global financial and economic uncertainties have significant negative effects on local industrial production, employment, and the stock market, while we find hardly any influence of local uncertainty on these variables. In addition we perform a forecasting analysis, where we assess the merits of uncertainty indicators for forecasting industrial production, employment and the stock market, using different performance measures. The results suggest that financial uncertainty significantly improves the forecasts of the stock market in terms of profit-based measures, while economic uncertainty gives, in general, more insight when forecasting macroeconomic variables.

## Introduction

In the aftermath of the 2008 financial crisis and the Great Recession, the interest of economists and policymakers has been markedly focused on the analysis of tools and techniques to assess the strengths and vulnerabilities of financial systems and, in particular, on measuring financial uncertainty and its effect on the economy. Also before the crisis, however, episodes of financial instability had highlighted the importance of continuous monitoring of financial systems in order to prevent crises. The International Monetary Fund, for example, had identified a broad set of prudential and macroeconomic variables that are relevant for assessing financial soundness (see International Monetary Fund 2002), which was later reduced to a subset including both aggregate bank balance sheet and income statement information and aggregate indicators of financial fragility of nonfinancial firms and nonbank financial markets. These indicators are referred to as financial soundness indicators and have, more recently, been examined with respect to their ability to predict financial sector distress (see Pietrzak 2021). The European Central Bank (ECB) has introduced a family of composite indicators of systemic stress (CISS) which are based on five categories—the financial intermediaries sector, money markets, equity markets, bond markets and foreign exchange markets—and which are supposed to measure a country’s financial stability.^1^

Other indicators which are (closely) related to the indicators of financial stability are so-called uncertainty indices. Because uncertainty is unobserved, a number of proxies have been proposed in the literature. Traditional methods include, for example, the disagreement among professional forecasters, see Zarnowitz and Lambros (1987) and Bomberger (1996). Another measure of financial uncertainty, which has become very popular, is the realized and implied stock market volatility, see Bloom (2009). A big advantage of this measure is that realized volatility, based on observed stock market returns, is readily available for almost all countries.

More recently, alternative measures using a more formal econometric framework have been introduced. Jurado et al. (2015) suggest that uncertainty relates to whether the economy is more or less predictable, i.e., less or more uncertain. The authors propose to use as uncertainty measure the common variation in forecast errors for a broad range of macroeconomic and financial variables. Rossi and Sekhposyan (2015) agree with Jurado et al. (2015) that uncertainty relates to whether the economy is more or less forecastable. The uncertainty index they propose is the percentile in the historical distribution of forecast errors associated with the realized forecast error. They distinguish between upside and downside uncertainty, because these uncertainties may affect the economy in different ways. Carriero et al. (2018) deal with common variation in the residual volatilities in a large vector autoregression model and estimate measures of uncertainty jointly with assessing its impact on the macroeconomy. Chuliá et al. (2017) propose an index of time-varying stock market uncertainty. The index is constructed by first removing the common variations in the series, based on identifying expected variation (risk) and unexpected variation (uncertainty). Baker et al. (2016) develop an index of economic policy uncertainty based on the frequency of key uncertainty-related terms that occur in newspaper articles. Böck et al. (2021) examine the merits of sovereign CDS volatility as an indicator of economic policy uncertainty, which, however, is not available for all countries. Scotti (2016) uses “surprises” from Bloomberg forecasts to construct measures of economic uncertainty. In contrast to most measures of uncertainty, which deal with common shocks, Bijapur (2021) proposes an indicator of firm-level uncertainty, which is composed of idiosyncratic shocks. Bloom (2014) surveys related literature.

Interest in financial and economic uncertainty has been spurred by a growing body of evidence that uncertainty rises sharply in recessions. In most of the literature, measures of uncertainty are estimated in a first step and then used as if they were observable data series in the following econometric analysis of its impact on macroeconomic variables. Most of the above cited studies include at least a small analysis on the effects of uncertainty on the economy. The authors include their preferred uncertainty measure, together with a small set of macroeconomic variables like industrial production, inflation and employment, in a vector autoregression model and examine the responses of the macroeconomic variables to the uncertainty shock. Uncertainty usually rises in economic downturns; but is uncertainty a source of business cycles or is it rather an endogenous response to them, and does the type of uncertainty matter? Ludvigson et al. (2021) find that higher macroeconomic uncertainty in recessions is often an endogenous response to output shocks, while financial uncertainty is a likely source of output fluctuations.

We propose to use financial and economic uncertainty indicators in the spirit of Jurado et al. (2015) in order to measure financial and economic (in)stability in the euro area, Germany, France, the United Kingdom and Austria. We thus follow the approach to remove the forecastable component of the variation of the variables under consideration and focus on the conditional expectation of the squared forecast errors. The data we use to compute our financial uncertainty index cover the main financial market segments: money market, equity market, (sovereign) bond market, and foreign exchange market. These data are available at a daily frequency and we transform them to monthly data (using monthly averages), because we propose to estimate financial uncertainty at a monthly frequency. The data we use to estimate our economic uncertainty index include sentiment indicators, data on employment, retail sales, manufacturing, orders, price indices, and survey data related order books, production expectations, employment expectations, etc. We construct both financial and economic uncertainty indices for the same countries, and examine the resulting differences.

First we assess the impact of both local (country specific) and global (US) financial and economic uncertainty on the economy by estimating a vector error correction (VEC) model and analysing the responses of main macroeconomic variables (industrial production, employment) and the stock market to a shock in uncertainty. Furthermore we examine the role of both local and global financial and economic uncertainty indices in forecasting. We also consider the ECB’s new composite indicator of systemic stress (CISS) as an alternative measure of financial instability. We use different VEC models including or excluding uncertainty indices and assess the respective forecasts. In doing so we employ both traditional loss-based performance measures (root mean squared error and mean absolute error) and profit-based measures (directional accuracy/hit rate and directional value).

The remainder of this paper is organized as follows. Section 2 revises the methodology used to estimate uncertainty. Section 3 describes the data and presents the resulting indices of financial and economic uncertainty. Section 4 describes the impulse response analysis and the forecasting analysis, and presents the corresponding results. All analyses are performed for the euro area, Germany, France, the United Kingdom and Austria. Section 5 summarizes and concludes.

## Methodology

Econometric studies on measuring uncertainty and its effects on the economy started with the seminal paper by Bloom (2009). Other relevant contributions include, among others, Bachmann et al. (2013), Baker et al. (2016), Basu and Bundick (2017), Berger et al. (2016), Caggiano et al. (2014), Chuliá et al. (2017), Carriero et al. (2018), Gilchrist et al. (2014), Jurado et al. (2015), and Scotti (2016),Bloom (2014) surveys related work.

In order to formally assess uncertainty we follow the approach focusing on unforecastable components of the variation of variables under consideration (see, e.g., Carriero et al. 2018; Chuliá et al. 2017; Jurado et al. 2015, later referred to as JLN). Below we briefly sketch the approach used in JLN, where the notion of uncertainty is formalized as follows: Let $y_{jt} \in Y_t \equiv \{ y_{1t}, \ldots , y_{Nt} \}$ be a variable and let $Y_t$ be the set of variables describing a certain sector, e.g., the financial sector, where we intend to measure uncertainty. Its $h-$period ahead uncertainty, ${\mathcal {U}}_{jt}(h)$, is the conditional volatility of the purely unforecastable component of the future value of a given variable. Namely,

1 $$\begin{aligned} {\mathcal {U}}_{jt}(h)=\sqrt{{{\mathbb {E}}}\left[ \left( y_{j,t+h}- {{\mathbb {E}}}[y_{j,t+h} | I_t] \right) ^2 | I_t \right] } \end{aligned}$$

where $I_t$ is information available at *t*.^2^ If the expectation at *t* of the squared error in forecasting $y_{j,t+h}$ rises then uncertainty in the variable rises. Uncertainty in the whole sector, approximated by the elements of $Y_t$, is an aggregate of individual uncertainties

2 $$\begin{aligned} {\mathcal {U}}_{t}^Y(h)=\textrm{plim}_{N \rightarrow \infty } \sum _{j=1}^N w_j \, {\mathcal {U}}_{jt}(h) \equiv {{\mathbb {E}}}\left[ {\mathcal {U}}_{jt}(h) \right] \end{aligned}$$

with the aggregation weights $w_j$ and the implicit assumption that the law of large numbers holds. The econometric framework of JLN, which we adopt, is based on the following main steps:

(i) The conditional expectation of the forecast error in (1), and thus ${{\mathbb {E}}}[y_{j,t+h} | I_t]$,^3^ is approximated by forecasts of diffusion indices (common factors). Common factors are estimated from a large set of predictors, $x_{it}$, $i=1, \ldots , N^x$. The information (in more technical terms the $\sigma$-field) generated by these predictors is assumed to approximate $I_t$ as closely as possible. In addition we assume that the conditional expectation is linear in $x_{it}$, $i=1, \ldots , N^x$. The common factors will be treated as known later on. Forecasts of both real activity and financial returns can be substantially improved by augmenting best-fitting conventional forecasting equations with common factors estimated from large datasets (see Ludvigson and Ng 2007, 2009; Stock and Watson 2006, among others)
  3 $$\begin{aligned} y_{j,t+1}=\Phi _j^y(L) y_{jt}+\gamma _j^F(L) {\hat{\textbf{F}}}_t + \gamma _j^W (L) {\textbf{W}}_t + \nu _{j,t+1} \end{aligned}$$
  where $\Phi _j^y(L)$, $\gamma _j^F(L)$ and $\gamma _j^W(L)$ are finite-order polynomials in the lag operator *L*,^4^ and ${\hat{\textbf{F}}}_t$ is the $k_F-$dimensional vector of estimates of latent common factors of the predictors ${\textbf{X}}_t=(x_{1t}, \ldots , x_{N^xt})'$ available for the analysis, which thus have the following factor structure
  4 $$\begin{aligned} x_{it}=(\Lambda _i^F)' {\textbf{F}}_t+e_{it} \end{aligned}$$
  ${\textbf{F}}_t$ is the $k_F$-dimensional vector of latent common factors, $\Lambda _i^F$ is the $k_F$-dimensional vector of factor loadings and $e_{it}$ is the idiosyncratic error. The number of factors, $k_F$, is much smaller than the number of series $N^x$. Finally, the $k_W$-dimensional vector ${\textbf{W}}_t$ contains additional predictors such as squares of ${\hat{F}}_{1t}$ and factors in $x_{it}^2$ to capture possible nonlinearities and potential effects that conditional volatilities might have on $y_{jt}$.^5^ Time varying volatilities of $y_{j,t+1}$, the factors and additional predictors are allowed. The estimation of the factors uses the method of static principal components. Factors are selected on the basis of potential predictive power, see (Bai and Ng 2002, 2006, 2008).
(ii) The conditional expectation of the squared forecast errors in (1) is computed from a parametric stochastic volatility model for the one-step-ahead predictive errors for both $y_{jt}$ and the factors.^6^ The conditional volatility for $h>1$ steps ahead is computed recursively, and through this procedure additional unforecastable variation is created via time varying volatility in the errors of the predictor variables (factors). In more detail, when allowing for the autoregressive dynamics in the factors and introducing notation $Y_{jt} \equiv \left( y_{jt},y_{jt-1},\dots ,y_{jt-q+1} \right) ' \in {\mathbb {R}}^q$, ${\textbf{Z}}_t \equiv \left( \hat{{\textbf{F}}}_t', {\textbf{W}}_t' \right) ' \in {\mathbb {R}}^{k}$, where $k= k_F+k_W$, and ${\mathcal {Z}}_t \equiv \left( {\textbf{Z}}_t', \ldots , {\textbf{Z}}_{t-q+1}' \right) ' \in {\mathbb {R}}^{kq}$, then we can obtain forecasts using the companion form
  5 $${\mathcal {Y}}_{jt} \equiv \underbrace{ \left[ \begin{array}{l} {\mathcal {Z}}_t \\ Y_{jt} \end{array} \right] }_{(k+1)q \times 1} = \left[ \begin{array}{ll} \underbrace{ \Phi ^{{\mathcal {Z}}} }_{kq \times kq} & \underbrace{0}_{kq \times q} \\ \underbrace{\Lambda_j^{\prime } }_{q \times kq} & \underbrace{\Phi _j^Y}_{q \times q} \end{array} \right] \left[ \begin{array}{c} {\mathcal {Z}}_{t-1} \\ Y_{j,t-1} \end{array} \right] + \left[ \begin{array}{c} {\nu }_t^{{\mathcal {Z}}} \\ {\nu }_{jt}^{Y} \end{array} \right]$$
  where $\Lambda _j$ and $\Phi _j^Y$ are functions of the coefficients in the lag polynomials in (3) and $\Phi ^{{\mathcal {Z}}}$ records coefficients of the components in ${\mathcal {Z}}_t$. In addition, stationarity of the corresponding time series is assumed.^7^ Let $\Omega _{jt}(h) \equiv {{\mathbb {E}}}_t \left( {\mathcal {Y}}_{j,t+h}-{{\mathbb {E}}}_t \left( {\mathcal {Y}}_{j,t+h} \right) \right) \left( {\mathcal {Y}}_{j,t+h}-{{\mathbb {E}}}_t \left( {\mathcal {Y}}_{j,t+h}\right) \right) '$, be the forecast error variance of ${\mathcal {Y}}_{jt}$ modelled in (5) which evolves as
  6 $$\begin{aligned} \Omega _{jt}(1)= {{\mathbb {E}}}_t \left( {\mathcal {Y}}_{j,t+1}^{\nu } \left( {\mathcal {Y}}_{j,t+1}^{\nu } \right) ' \right) \end{aligned}$$
  and
  7 $$\begin{aligned} \Omega _{jt}(h)= \Phi _j^{{\mathcal {Y}}} \left[ \Omega _{jt}(h-1) \right] \left( \Phi _j^{{\mathcal {Y}}} \right) ' + {{\mathbb {E}}}_t \left( {\mathcal {Y}}_{j,t+h}^{\nu } \left( {\mathcal {Y}}_{j,t+h}^{\nu } \right) ' \right) \ \ \textrm{for} \ \ h>1 \end{aligned}$$
  see Eq. (9) in JLN, where ${\mathcal {Y}}_{j,t}^{\nu }=\left( ({\nu }_t^{{\mathcal {Z}}})', ({\nu }_{jt}^{Y})' \right) '$ and
  8 $$\begin{aligned} \Phi _j^{{\mathcal {Y}}}= \left[ \begin{array}{cc} \Phi ^{{\mathcal {Z}}} & 0 \\ \Lambda _j^{\prime } & \Phi _j^Y \end{array} \right] \end{aligned}$$
  Thus, the expected forecast uncertainty of $y_{j,t+h}$ is the square root of the corresponding scalar on the diagonal of $\Omega _{jt}(h)$, i.e.,
  9 $$\begin{aligned} {\mathcal {U}}_{jt}(h)= \sqrt{e_j' \, \Omega _{jt}(h) e_j} \end{aligned}$$
  where $e_j$ is the corresponding selection vector. In addition, stochastic volatility of $y_{jt}$ and the factors is assumed, i.e., $\nu _{j,t+1}=\sigma _{j,t+1} \varepsilon _{j,t+1}$ with $\varepsilon _{j,t+1} \sim N(0,1)$ and
  10 $$\begin{aligned} \log \left( \sigma _{j,t+1}^2 \right) = \alpha _j + \beta _j \log \left( \sigma _{jt}^2 \right) + \tau _j \eta _{j,t+1}, \ \ \eta _{j,t+1} \sim N(0,1) \end{aligned}$$
  which affects the time variation in uncertainty (7) (see JLN, page 1187). Equation (10) can be estimated using Markov Chain Monte Carlo (MCMC) methods, following Kastner and Frühwirth-Schnatter (2014) and Kastner (2016).
(iii) The aggregate uncertainty, ${\mathcal {U}}_{t}^Y(h)$, is estimated from individual uncertainty measures ${\mathcal {U}}_{jt}(h)$. We consider two kinds of weights: equal weights and weights based on the common factors in the individual measures of uncertainty. As the implied uncertainty indices are very similar, we use the simpler version based on equal weights in this paper.

We use slightly modified versions of the codes provided by Jurado et al. (2015) to compute our financial and economic uncertainty indices.

## Data and uncertainty indices

The following subsections describe the data used for estimating the uncertainty indices and present graphs of the estimated financial and economic uncertainty indices, for the euro area (EA), Germany (DE), France (FR), the United Kingdom (UK), and Austria (AT).

### Data

The financial data we use in order to estimate the financial uncertainty index include monthly observations on interest rates, yields on government bonds, yields on corporate bonds, interest rate swaps, overnight interest rates, spreads between different yields and/or rates, stock indices, bond indices, foreign exchange rates, dividend-price ratios, earnings-price ratios, and volatilities of stock/bond indices and foreign exchange returns. We consider different maturities for the rates/yields and use averages of the daily observations to compute monthly values. In total we have 74 financial variables for the euro area and Germany, 72 for France, 76 for the UK, and 77 for Austria, when we compute the financial uncertainty indices. The data set which is used to extract the factors used for forecasting the conditional volatilities for the financial variables, consists of both the financial variables just described and additional macroeconomic variables. The macroeconomic variables include sentiment indicators, data on employment, retail sales, manufacturing, orders, price indices, and survey data for twelve industries related to important economic questions concerning order books, production trend observed in recent months, production expectations, employment expectations, etc.^8^ Note that the macroeconomic data are not real-time but ex-post (possibly revised) time series.^9^ The macroeconomic data set includes 122 time series for the euro area and Austria, 120 for Germany, and 114 times series for France and the UK, respectively.^10^ All data range from January 2000 until December 2020, i.e., we have 252 observations per variable. Details on the data used and a list of all variables considered for the euro area can be found in Appendix A. When we compute the macroeconomic uncertainty indicator we use again the macroeconomic and the financial data to extract the factors, but we forecast conditional volatilities for the macroeconomic variables (not for the financial variables). In doing so we follow Jurado et al. (2015) to group some variables which are originally included in the financial variables with the macroeconomic variables. In this case $N=135$ for the euro area, $N=134$ for Germany, $N=128$ for France and the UK, respectively, and $N=138$ for Austria.

In a prior version of this article we also use aggregate banking data to construct alternative versions of the financial uncertainty indices (for the euro area and Austria), in order to detect potential differences and, in particular, to analyze whether banking data improve the predictive properties of financial uncertainty. We find that, overall, banking data do not seem to improve the forecast performance. For more details, see Fortin et al. (2021).

### Uncertainty indices

**Fig. 1:**
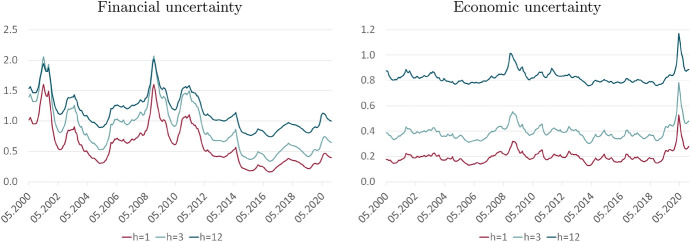
Financial and economic uncertainty indices for the euro area, for forecast horizons of one, three and twelve months

**Fig. 2:**
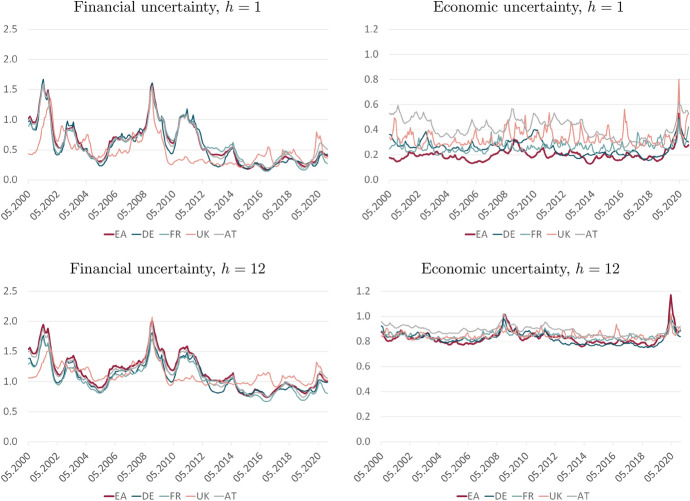
Financial and economic uncertainty indices for the euro area, Germany, France, the United Kingdom and Austria, for forecast horizons of one (top row) and twelve (bottow row) months

Figure 1 presents the financial and economic uncertainty indices for the euro area, showing three indices in each case, relating to forecast horizons of one, three and twelve months. While the level of uncertainty clearly increases with the forecast horizon (on average), the variability of uncertainty decreases, at least with the larger forecast horizon of twelve months.^11^ This is also true for the country specific uncertainty indices, see Fig. 2, which shows the country indices for financial and economic uncertainty, for forecast horizons of one and twelve months. Financial uncertainty indices in the euro area, Germany, France and Austria show very similar developments and reveal spikes around the bursting of the dot-com bubble 2000–2001, the global financial crisis 2007–2008, the European sovereign debt crisis 2010–2011, as well as around the outbreak of the Covid-19 crisis in early 2020. The UK is a bit different. The European sovereign debt crisis 2010–2011 is obviously not reflected in the UK financial uncertainty index, the most pronounced spike here corresponds to the global financial crisis (2007–2008), and the peak around the Covid-19 crisis is larger than in other countries. In all countries and the euro area economic uncertainty exhibits both a smaller level (on average) and a significantly smaller variability than financial uncertainty. Economic uncertainty in the euro area exhibits two peaks, one around the global financial crisis (great depression) and one around the outbreak of the Covid-19 crisis in 2020. Albeit rather similar, the development of economic uncertainty is more diverse among the countries than that of financial uncertainty.^12^ In particular the spikes around the global financial crisis are not so clearly pronounced in all countries. Further, the Covid-19 crisis suggests an exceptionally large increase in economic uncertainty in the UK, like for financial uncertainty, for a forecast horizon of one month, and there is another peak in the UK after the Brexit referendum in June 2016.

The financial uncertainty indices for different forecast horizons, for the euro area and the countries, are highly correlated (above 0.96). This is true across different forecast horizons, and also across the regions, if the UK is not considered. Clearly, UK financial uncertainty is not so highly correlated with financial uncertainty in the other countries or the euro area (around 0.6). Also the economic uncertainty indices are positively correlated within the countries (above 0.65 and mostly larger), however, at a lower degree across the countries (0.2$-$0.7). With economic uncertainty the correlation across countries increases with the forecast horizon, in particular for euro area countries, see Fig. 2. The descriptive statistics suggest that all uncertainty indices exhibit a (strongly) positive skewness.^13^ This implies that the distribution is not symmetric and, in particular, that the right tail of the distribution is longer and the mass of the distribution is concentrated on the left. The kurtosis is mostly around three, which is the value for the Gaussian distribution, only for economic uncertainty in the euro area and the UK the numbers are around/above ten. This suggests that the underlying distribution produces more extreme realizations than the normal distribution. When looking at Figs. 1 and 2 we observe particularly sharp increases in economic uncertainty during the Covid-19 crisis for the euro area and the UK. This might be one of the drivers of excess kurtosis for economic uncertainty. Indeed, when estimating the kurtosis of economic uncertainty for the subsample excluding the Covid-19 crisis (May 2000 to December 2019), we obtain values which are much lower than for the total sample.

## Empirical analysis

The data sample covers monthly observations for the period ranging from May 2000 through December 2020. We do not start earlier because our uncertainty indices can only be created from May 2000 onwards, due to data availability of the predictors and the autoregressive structure of (3), where the number of lags is four. For all countries under consideration we perform an impulse response analysis to quantify the dynamic responses of macroeconomic variables (industrial production, employment) and stock market indices to uncertainty shocks (of both financial and economic nature). We use the Cholesky decomposition to identify the structural shocks, in the vector error correction (VEC) model

11 $$\begin{aligned} \Delta {\textbf{y}}_{t}= & {\textbf{c}} + {\varvec{\alpha }}{{\varvec{\beta }}}' {\textbf{y}}_{t-1} + \sum _{j=1}^{p} {\mathbf {\Gamma }}_{j} \Delta {\textbf{y}}_{t-j} + {\textbf{u}}_{t} \ \ \ \ \end{aligned}$$

where $\left( {\textbf{y}}_{t} \right)$ is an $n-$dimensional stochastic process, *t* denotes the time dimension, and ${\textbf{c}}$ is an $n-$dimensional vector of intercept terms. The parameter matrix ${\varvec{\alpha }}$ is of dimension $n \times r$, while the matrix of cointegrating vectors ${\varvec{\beta }}$ is an $n \times r$ matrix, where *n* is the number of variables and *r* is the number of cointegrating relationships. For matrix ${\varvec{\beta }}$ we apply the usual normalization such that ${\varvec{\beta }}_{1:r,1:r}$ is the *r*-dimensional identity matrix. The short-run dynamics are described by the $n \times n$ matrices ${\varvec{\Gamma }}_{j}$, $j=1,\dots ,p$. Finally, ${\textbf{u}}_{t}$ is a white noise process with mean zero and covariance matrix $\varvec{\Sigma }$.^14^

Our VEC model contains the following (endogenous) variables: the *global* (US) financial or economic uncertainty index, $x_t^{US}$, the corresponding *local* (country specific) uncertainty index, $x_t^{j}$, and the country specific variables: industrial production, $ip_t^{j}$, employment, $empl_t^{j}$, the consumer price index, $cpi_t^{j}$, the short-term interest rate, $ir_t$, and the stock market index, $stm_t^j$, where $j \in \left\{ \text {EA}, \text {DE}, \text {FR}, \text {UK}, \text {AT} \right\}$, $ir=$ 3 m-Euribor for euro area countries, $ir=$ 3 m-Libor for the UK, and the stock market indices are the Euro Stoxx 50, the DAX 30,^15^ the CAC 40, the FTSE 100 and the ATX. Hence, $n=7$ and ${\textbf{y}}_t^j= \left( x_t^{US}, x_t^{j}, ip_t^{j}, empl_t^{j}, cpi_t^{j}, ir_t^j, stm_t^j \right) '$. All variables except the uncertainty indices and the interest rate enter in log levels. To describe global (financial and economic) uncertainty we use the US financial and economic uncertainty indicators as calculated by Jurado et al. (2015).^16^ For all financial and economic uncertainty indices we use the one-month ahead uncertainties.

The number of lags *p* is chosen based on the Schwarz information criterion.^17^ The application of the error correction model (11) is supported as follows: For the time series considered, except for the uncertainty indices, the null hypothesis of a unit root cannot be rejected at the 5% significance level, using augmented Dickey-Fuller tests. To deal with stationary variables in the VEC model we follow Lütkepohl (2005)[page 250] on the restrictions of cointegrating vectors in ${\varvec{\beta }}$. That is, the first two coordinates of ${\textbf{y}}_t$ are global and local (stationary) uncertainty indices, $x_t^{US}$ and $x_t^j$, and thus the first two cointegrating vectors are the corresponding canonical basis vectors.^18^ We perform Johansen cointegration tests among all integrated endogenous variables and obtain evidence of one additional cointegrating vector, for each country *j* and the euro area. Thus, we have three cointegrating vectors, i.e., ${\hat{r}}=3$.

In the current specification of the VEC model (11) all the variables considered are assumed to be endogenous. Assuming that the model is correctly specified, the parameters can be estimated consistently (see, e.g., Lütkepohl 2005). Note that the current specification allows to estimate the impact and the reverse impact of the global uncertainty index, approximated by $x_t^{US}$, and of the local uncertainty index, $x_t^j$. At least for larger countries and the euro area, effects in both directions cannot be ruled out a priori. Therefore, also the global uncertainty index is modelled endogenously within our VEC model. To investigate the stability of our modelling approach, as a robustness check, we include the S &P 500 index or US industrial production as exogenous variables.^19^ When comparing the impulse response functions of (11) with those obtained when including US industrial production or the S&P 500 index, we see that the differences are neglectable, which supports to proceed with the VEC model as defined in (11).^20^

### Impulse response analysis

**Fig. 3:**
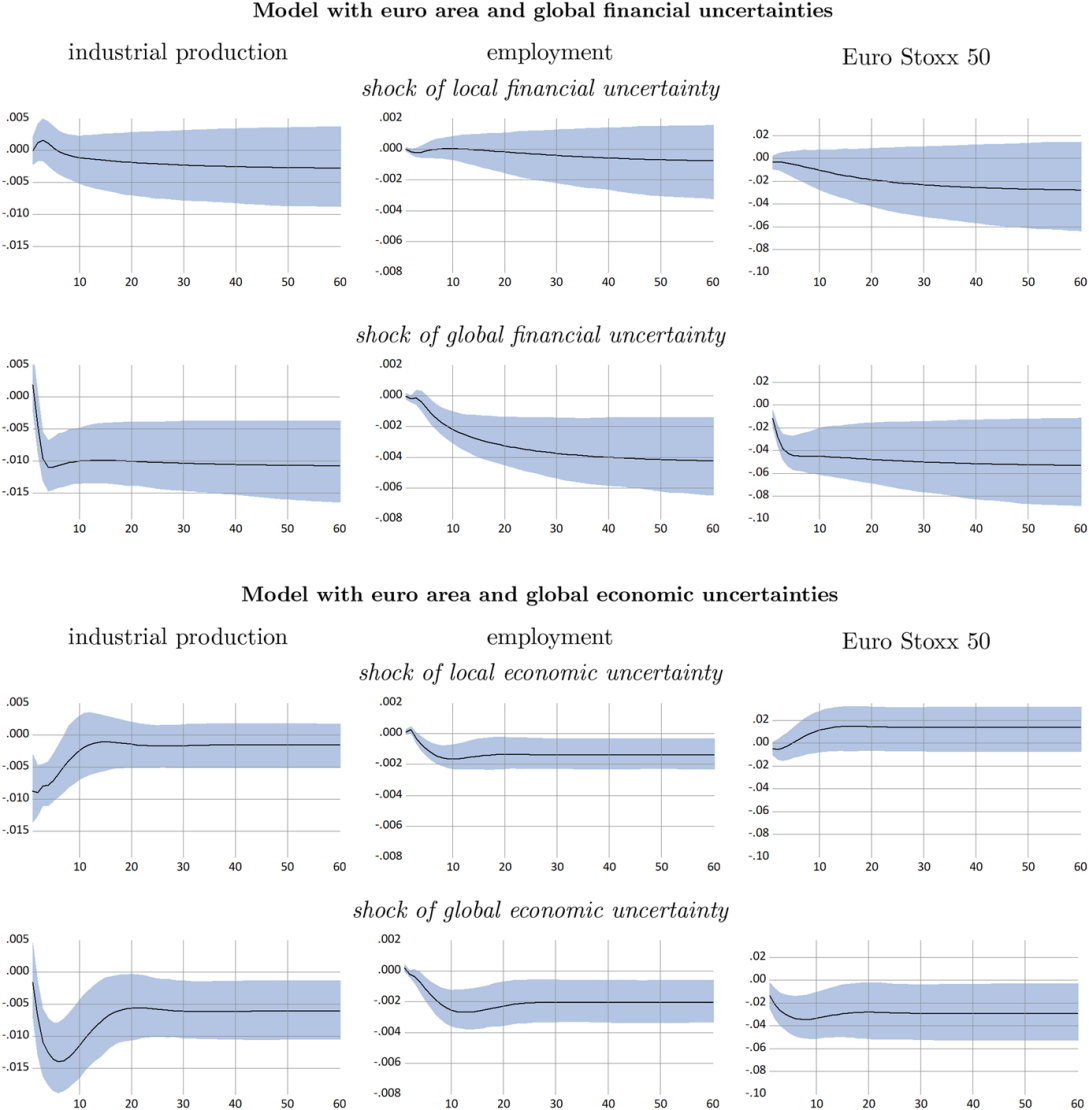
Impulse responses of industrial production, employment and the Euro Stoxx 50 to a one standard deviation shock of financial uncertainty (first block) and of economic uncertainty (second block) for the euro area and $h=1$, with 95% confidence intervals. In each block the first row shows the effect of euro area (financial/economic) uncertainty, the second row shows the effect of global (financial/economic) uncertainty

To identify the impact of an uncertainty shock on macroeconomic variables and the stock market we employ an impulse response analysis based on the Cholesky decomposition. We present results of estimated impulse responses of logged values of industrial production, employment and the stock market to one standard deviation increases (“shocks”) of either the financial or the economic uncertainty index, over the next 60 months, where we consider both global and local indices, respectively.^21^ Fig. 3 shows the graphs for the euro area, Figs. 6, 7, 8 and 9 (in Appendix B.1) present results for the other countries.

The first row of graphs in Fig. 3 shows the estimated impact of an increase in the euro area *(local) financial* uncertainty. Given the 95% confidence bounds shown by the shaded areas, we do not observe any significant impact of local financial uncertainty on industrial production and employment, nor on the Euro Stoxx 50. The second row shows the effects of an increase in *global financial* uncertainty. In this case, contrary to the situation before, we do see statistically significant decreases of industrial production, employment and the stock market. Global financial uncertainty thus seems to be a more influential factor for economic activity and the stock market than local financial uncertainty. The third row considers the impact of an increase in *local economic* uncertainty, and we observe a significant albeit short-run decrease for industrial production, while for employment we see a small and significant permanent decline; however, there is no significant impact of local economic uncertainty on the stock market. Finally, the fourth row considers the impact of an increase in *global economic* uncertainty, and here we see statistically significant negative effects upon all three variables considered.

To summarize, the impact of global uncertainty, both financial and economic, on euro area industrial production, employment and on the Euro Stoxx 50 is always significant, while this is never the case for local financial uncertainty. Note that the impact of global *financial* uncertainty exceeds the impact of global *economic* uncertainty. However, the impact of local economic uncertainty on the macroeconomic variables is also significant, albeit much smaller in size and persistence than that of global economic uncertainty. Somewhat surprisingly, although local uncertainty indices are constructed on the basis of local data, our impulse response analysis mainly identifies global uncertainty as a key driving factor of economic and financial activity.

**Fig. 4:**
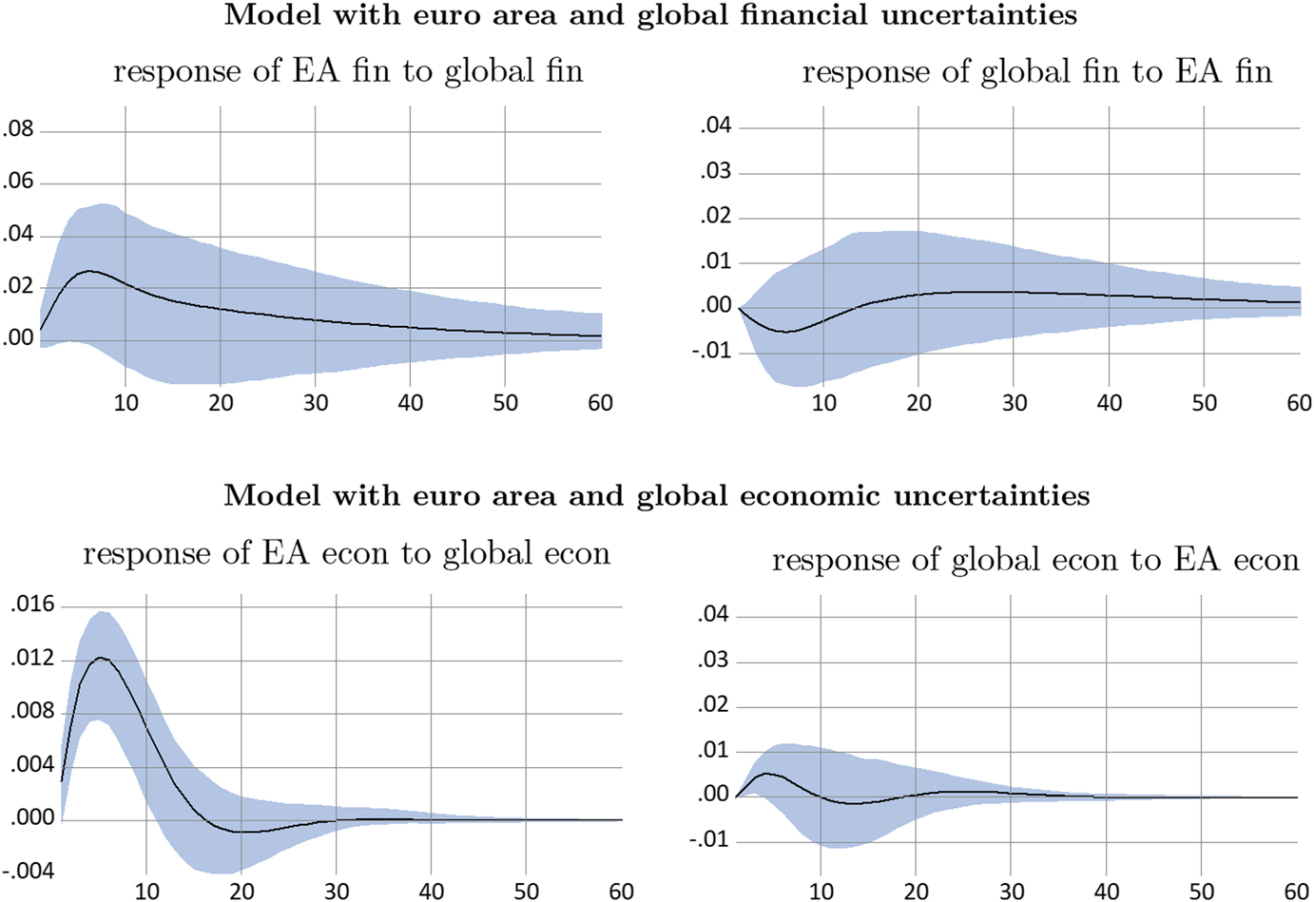
Effect of global uncertainty on euro area uncertainty and the other way round. The graphs show the effect of global uncertainty upon euro area uncertainty (left) and the effect of euro area uncertainty upon global uncertainty (right). The first row shows the case for financial uncertainties, the second row shows the case for economic uncertainties

As local and global uncertainty indicators enter the corresponding VEC models we can also investigate how local and global uncertainty indices influence each other. Interestingly we observe a strong significant impact of global on local *economic* uncertainty for approximately 2.5 years, while the impact of global on local *financial* uncertainty is barely significant, and only observable for about a year, see Fig. 4. However, we hardly see any significant impact of euro area uncertainty upon global uncertainty, neither for financial nor for economic uncertainty, although this could possibly be the case for a large area like the euro area.

Looking at individual countries (see Figs. 6, 7, 8 and 9 in Appendix B.1), the impulse response results are roughly similar as for the euro area. Local stock markets seem to be influenced mainly by global uncertainty. Both global financial and global economic uncertainty show significant effects on all local stock markets, where the impact of financial uncertainty is found to be stronger (in magnitude and/or persistence). Note that the long-term impact of global financial uncertainty upon local stock markets is largest for Austria and smallest for the UK. On the other hand, local uncertainty (neither financial nor economic) does not seem to be an important factor for stock markets. Among all countries considered (including the euro area), only in the UK the local stock market responds significantly, albeit only very shortly (four quarters), to a shock in local financial uncertainty.

Also country specific industrial production seems to be influenced more by global than by local uncertainty, where the magnitude of the effect is usually larger for global *economic* than global financial uncertainty (except for France and Austria). Local financial uncertainty does never significantly impact industrial production; local economic uncertainty, however, shows a significant but short-term effect for Germany (six quarters) and the UK (two quarters). The impulse response results for employment are very similar.

### Forecasting analysis

To analyze the value added of our uncertainty indices for forecasting industrial production, employment and the stock market, we compare the forecast performance of the VEC models forecasting these variables when the uncertainty indices are included and when they are omitted. In addition, we consider the forecast performance of a VEC model when the uncertainty index is replaced by the CISS, and we examine two benchmark models, the random walk (RW) and the univariate autoregressive model of order one, DAR(1).^22^

We consider rolling-window estimation for our analysis, i.e., we keep the size of the estimation sample constant and equal to eighty months, and move forward the sample by one month, re-estimating the model parameters. The out-of-sample period, in which we evaluate the forecast performance, ranges from January 2007 to December 2020. In order to evaluate different forecasts we do not only employ traditional loss measures, like root mean squared error (RMSE) and mean absolute error (MAE), but also profit-based measures like directional accuracy (DA) and directional value (DV). The directional accuracy, or hit rate, is a binary variable measuring whether the direction of a variable change was correctly forecasted. The directional value additionally incorporates the economic value of directional forecasts by assigning to each correctly predicted change its magnitude. The loss-based and profit-based performance measures are formally defined as follows

$$\begin{aligned} AE_{t+h,h}= \,& \Big | {\hat{z}}_{t+h|t} - z_{t+h} \Big | \\ SE_{t+h,h}= \,& \left( {\hat{z}}_{t+h|t} - z_{t+h} \right) ^2 \\ DA_{t+h,h}= \,& {\mathbb {I}} \left( \text{ sgn }(z_{t+h}-z_{t})=\text{ sgn }({\hat{z}}_{t+h|t}-z_{t})\right) \\ DV_{t+h,h}= \,& \left| z_{t+h}-z_t\right| \; DA_{t+h,h} \end{aligned}$$

where $z_{t}$ is the variable we want to forecast, namely $z_t \in \left\{ ip_t^j, empl_t^j, stm_t^j \right\}$ at time *t*, for country $j \in \left\{ \text {EA}, \text {DE}, \text {FR}, \text {UK}, \text {AT} \right\}$, and ${\hat{z}}_{t+h|t}$ is the forecast of the variable for time $t+h$ conditional on the information available at time *t*, i.e., *h* is the forecast horizon, and ${\mathbb {I}} (\cdot )$ is the indicator function. The aggregate performance measures for each model are calculated over the out-of-sample period for a given forecast horizon as follows

$$\begin{aligned} RMSE_h= & 100\sqrt{\sum _{j=0}^{T_2-T_1} \frac{SE_{T_1+j,h}}{T_2-T_1+1}} \\ MAE_h= & 100\sum _{j=0}^{T_2-T_1} \frac{AE_{T_1+j,h}}{T_2-T_1+1} \\ DA_h= & 100 \sum _{j=0}^{T_2-T_1} \frac{DA_{T_1+j,h}}{T_2-T_1+1} \\ DV_h= & 100 \, \frac{\sum _{j=0}^{T_2-T_1} DV_{T_1+j,h}}{\sum _{j=0}^{T_2-T_1} |z_{T_1+j}-z_{T_1+j-h}|} \\= & 100 \, \frac{\sum _{j=0}^{T_2-T_1} |{\hat{z}}_{T_1+j | T_1+j-h}-z_{T_1+j-h}| DA_{T_1+j,h}}{\sum _{j=0}^{T_2-T_1} |z_{T_1+j}-z_{T_1+j-h}|} \end{aligned}$$

where $T_1=$ January 2007 and $T_2=$ December 2020. We compare the forecast performance of the VEC models for the cases with: (i) both local (country driven) and global (US) financial uncertainty indices, (ii) both local and global economic uncertainty indices, (iii) the local financial uncertainty index, (iv) the local economic uncertainty index, (v) the country specific CISS,^23^ (vi) no uncertainty and no CISS indices, and for two benchmark models,^24^ (vii) autoregressive model of order one in differences, DAR(1), and (viii) random walk (RW). We consider forecast horizons of one and twelve months.

#### Euro area

Table 1 presents the forecast performance of the different models described above for the euro area. The first, second and third blocks present the forecast performance for industrial production, employment and the stock market, respectively. When forecasting industrial production, the best performance regarding loss measures (RMSE and MAE) is achieved by the random walk for both forecast horizons. With respect to profit-based measures and a forecast horizon of one month, the best hit rate is implied by the model including the CISS, while the best directional value (DV) is achieved by the model with both local and global economic uncertainty indices. For a forecast horizon of twelve months the best model with respect to the hit rate is the one with local economic uncertainty and with respect to the directional value it is the model with the CISS.

Regarding the forecast performance for employment we observe that uncertainty indices improve the forecast performance regarding loss measures. For a forecast horizon of one month, the model with CISS gives the smallest RMSE and the model with both global and local financial uncertainty indices implies the smallest MAE (which, however, is only marginally lower than for other models). For a forecast horizon of twelve months, the model with local economic uncertainty yields the smallest RMSE, while the model with local financial uncertainty yields the smallest MAE. Regarding profit-based measures, the model with the CISS always performs best.

Finally, we observe the following pattern in the forecast performance for the Euro Stoxx 50. While benchmark models provide the lowest RMSE and MAE, the model with both local and global *financial* uncertainty yields the largest hit rate and directional value, for forecast horizons of one and twelve months.

**Fig. 5:**
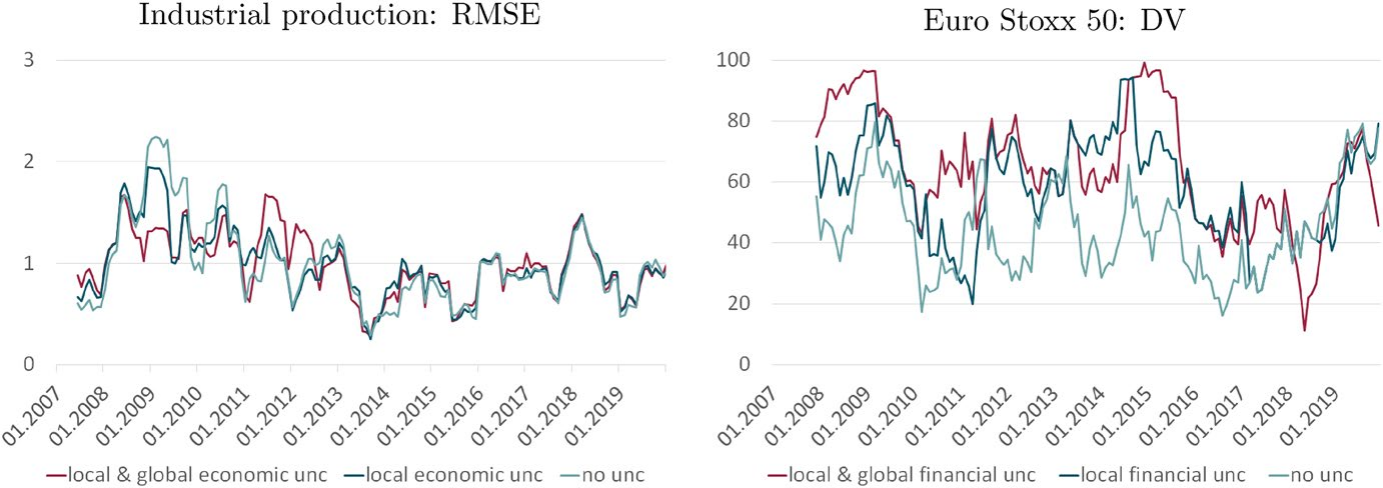
Forecasting industrial production and the Euro Stoxx 50 in the euro area with both local and global (economic/financial) uncertainties, with only local (economic/financial) uncertainty and with no uncertainty, for $h=1$. The RMSE and the DV are shown for rolling windows over six and twelve months, respectively

**Fig. 6:**
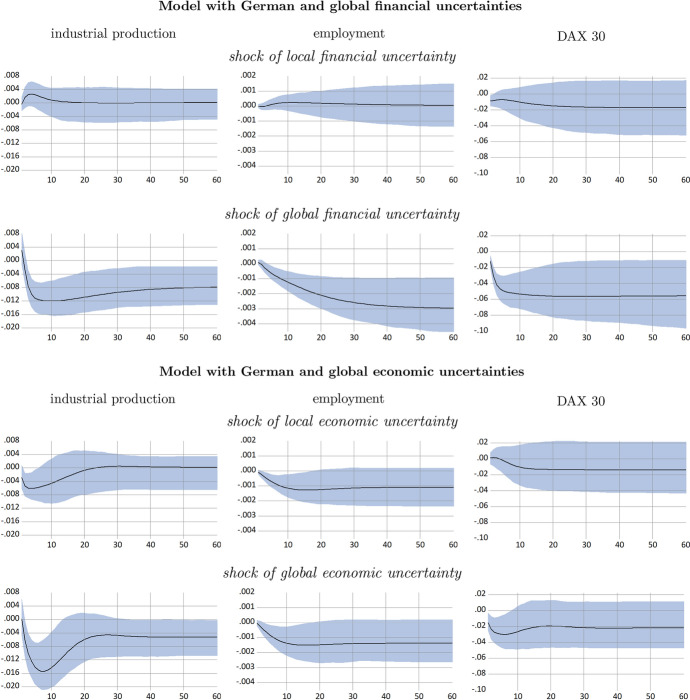
Impulse responses of industrial production, employment and the DAX 30 to a one standard deviation shock of financial uncertainty (first block) and of economic uncertainty (second block) for Germany and $h=1$, with 95% confidence intervals. In each block the first row shows the effect of German (financial/economic) uncertainty, the second row shows the effect of global (financial/economic) uncertainty

**Fig. 7:**
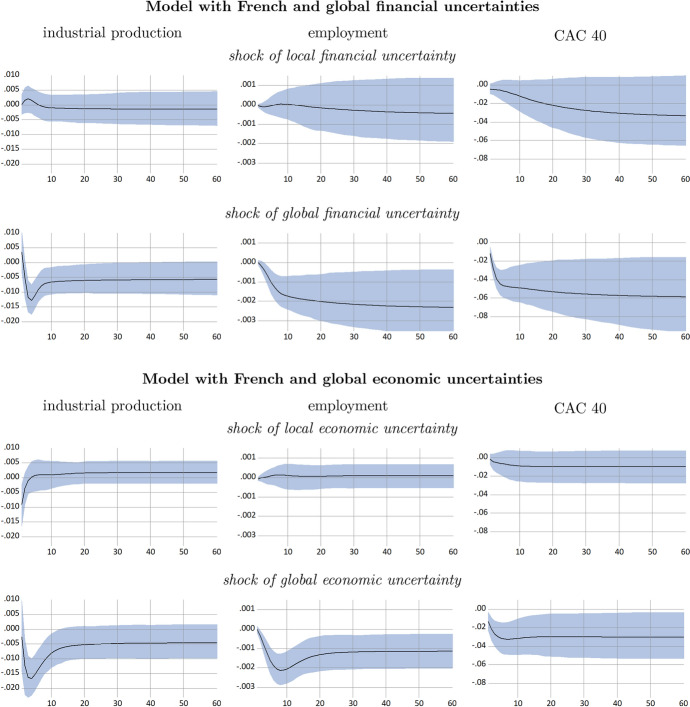
Impulse responses of industrial production, employment and the CAC 40 to a one standard deviation shock of financial uncertainty (first block) and of economic uncertainty (second block) for France and $h=1$, with 95% confidence intervals. In each block the first row shows the effect of French (financial/economic) uncertainty, the second row shows the effect of global (financial/economic) uncertainty

**Fig. 8:**
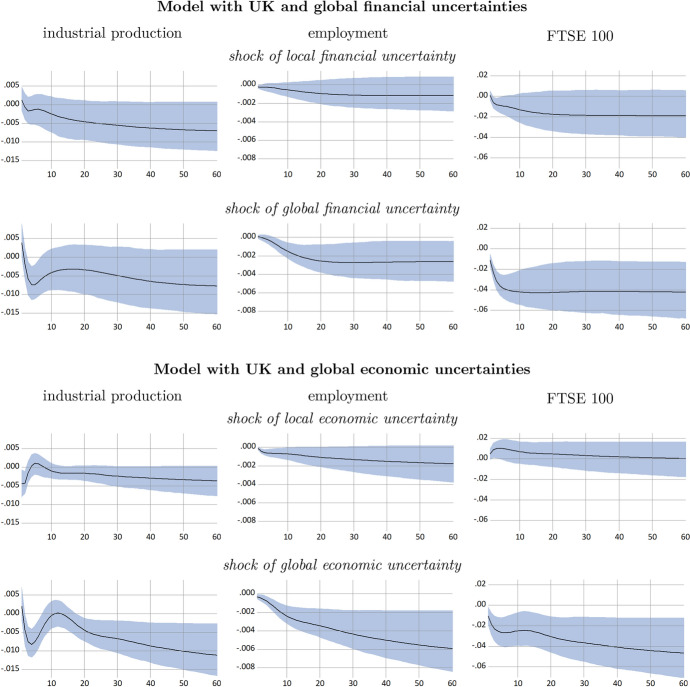
Impulse responses of industrial production, employment and the FTSE 100 to a one standard deviation shock of financial uncertainty (first block) and of economic uncertainty (second block) for the United Kingdom and $h=1$, with 95% confidence intervals. In each block the first row shows the effect of UK (financial/economic) uncertainty, the second row shows the effect of global (financial/economic) uncertainty

**Fig. 9:**
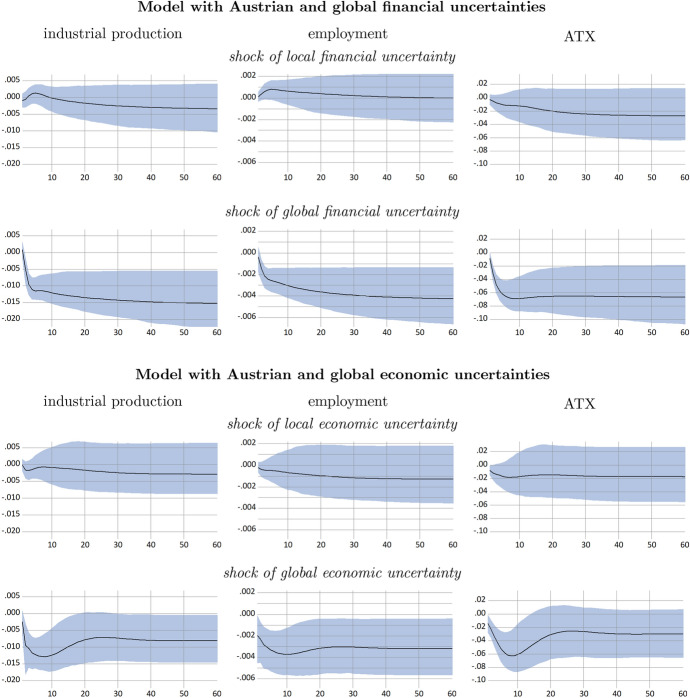
Impulse responses of industrial production, employment and the ATX to a one standard deviation shock of financial uncertainty (first block) and of economic uncertainty (second block) for Austria and $h=1$, with 95% confidence intervals. In each block the first row shows the effect of Austrian (financial/economic) uncertainty, the second row shows the effect of global (financial/economic) uncertainty

While Table 1 presents forecast performance criteria over the total out-of-sample period (January 2007–December 2020) it is also interesting to look at the forecast performance in sub-periods, to get an idea about which model performs best in which sub-period. Figure 5 shows the RMSE over rolling windows of six months for forecasting industrial production in the euro area, and the directional value over rolling windows of twelve months for forecasting the Euro Stoxx 50, for a forecast horizon of one month. For industrial production we show the time-changing RMSE implied by the models including both local and global *economic* uncertainties, including only local economic uncertainty and including no uncertainty. For the Euro Stoxx 50 we present the time-changing DV implied by the models including both local and global *financial* uncertainties, including only local financial uncertainty and including no uncertainty. Note that in the case of industrial production the model with both local and global economic uncertainties provides the lowest RMSE in the period of global financial crisis,^25^ while the same model yields the worst performance in the period of the European sovereign debt crisis. Thus considering global economic uncertainty improves forecasts during the global financial crisis but does not seem to be helpful in the euro area crisis. However, the model including both local and global financial uncertainties yields the largest DV for the Euro Stoxx 50 most of the time, including the period of the global financial crisis.

#### Other countries

We present the results related to the forecast performance for Germany, France, the UK and Austria in Tables 5, 6, 7 and 8 in Appendix B.2.

#### Industrial production

In all countries we observe that models including *economic* uncertainty provide the best forecast performance for industrial production in terms of the RMSE and DV, for a forecast horizon of one month. In addition, we observe that both local and global *financial* uncertainties are important in forecasting industrial production, especially in Germany and France. For Germany this model yields the largest hit rate for a forecast horizon of one month and the largest DV for a forecast horizon of twelve months, while for France this model implies the largest hit rate and DV for a forecast horizon of twelve months. To summarize, the models with both local and global economic or financial uncertainties yield the best profit-based performance when forecasting industrial production, over the short and the long forecast horizons for Germany and France, while for the UK and Austria models with only economic uncertainty improve the forecast performance for a forecast horizon of one month.

#### Employment

Unlike with industrial production, only models with economic (not financial) uncertainty provide the best forecast performance when forecasting employment. For Germany this is the case for the RMSE and for profit measures, for a forecast horizon of one month. For a forecast horizon of twelve months, however, the model with no uncertainty yields the best forecast performance with respect to all measures. For France the models with economic uncertainty yield the smallest RMSE and MAE over both horizons. For the UK models with economic uncertainty imply smallest loss measures only for the longer forecast horizon, but largest profit measures for both the short and long forecast horizons.^26^ For Austria the model with only local economic uncertainty implies the largest DV for a forecast horizon of one month. In all other cases the benchmark models yield the best performance. All in all, the models with both local and global *economic* uncertainty dominate the best models when forecasting employment. This holds for all countries but Austria, where the model with only local economic uncertainty seems to perform better.

#### Stock market

We observe a similar pattern in the forecast performance of stock market indices for Germany, France, the UK and Austria as in the euro area. Models with both local and global *financial* uncertainty yield the largest hit rates and directional values, while benchmark models provide the lowest RMSE and MAE, over both forecast horizons. For Austria, the model with both local and global financial uncertainty also yields the lowest RMSE, for a forecast horizon of one month.

In order to find out whether certain models forecast significantly better than others (with respect to a given performance measure), we perform the Diebold-Mariano test of equal forecast accuracy (see Diebold and Mariano 1995). We are particularly interested in whether models including uncertainty indices achieve significantly better forecasts than models without uncertainty indices. More precisely, we test whether the model including both local and global (financial or economic) uncertainties provides a significantly better forecast performance (at the 10% level) than the model with only local uncertainty^27^ or the model with no uncertainty. In addition we test whether the model including (only) local uncertainty provides better forecasts than the model with no uncertainty. Our main results are as follows. We find significant differences between models only when forecasting stock market indices, considering profit-based measures. First, the models including both local and global financial uncertainties significantly outperform, except for Germany, the models including no uncertainty for forecast horizons of one and twelve months.^28^ Second, models with both local and global financial uncertainties significantly outperform models with only local financial uncertainty, for a forecast horizon of one month.^29^ This implies that for short-term forecasting global uncertainty seems to be more important than local uncertainty. Third, for all countries but Germany, models with only local financial uncertainty significantly outperform models with no uncertainty, for a forecast horizon of twelve months. Local financial uncertainty thus seems to be more important when forecasting over longer than over short horizons.

**Table 1:** Forecasting industrial production, employment and the stock market in the euro area

	RMSE	MAE	DA	DV	RMSE	MAE	DA	DV
	$h=1$				$h=12$			
	*Industrial production, EA*							
Local & global financial unc.	3.22	1.32	50.60	57.38	8.53	5.44	46.50	40.16
Local & global economic unc.	2.96	1.23	54.76	**59.09**	9.94	5.98	45.86	40.21
Local financial unc.	3.49	1.31	55.95	45.50	8.35	5.11	**50.96**	41.90
Local economic unc.	3.76	1.43	51.19	56.55	8.82	5.35	48.41	36.03
CISS	3.38	1.30	**58.33**	51.59	11.32	6.15	48.41	**53.94**
No uncertainty	3.52	1.34	56.55	47.13	9.30	5.53	43.95	34.92
DAR(1)	3.71	1.30	44.05	32.72	7.02	4.39	40.13	22.27
RW	**2.50**	**1.12**	44.05	40.79	**6.40**	**3.84**	46.43	31.46
	*Employment, EA*							
Local & global financial unc.	0.25	** 0.11**	79.76	77.37	0.45	**0.22**	70.06	76.91
Local & global economic unc.	0.24	0.12	71.43	70.85	1.29	0.91	64.97	68.46
Local financial unc.	0.23	0.11	79.76	85.36	1.18	**0.78**	72.61	76.46
Local economic unc.	0.26	0.12	73.81	69.32	**1.16**	0.82	64.97	68.17
CISS	**0.22**	0.11	**82.74**	**91.63**	0.47	0.89	**75.80**	**80.45**
No uncertainty	0.23	0.11	**82.74**	87.02	1.23	0.88	67.52	69.59
DAR(1)	0.30	0.12	67.26	61.64	1.46	1.16	43.95	45.19
RW	0.24	0.11	51.19	51.91	1.25	1.07	55.36	64.56
	*Euro Stoxx 50*							
Local & global financial unc.	5.48	3.88	**60.71**	**66.08**	40.95	23.51	**52.23**	**60.63**
Local & global economic unc.	6.54	4.38	53.57	49.43	45.43	22.00	51.59	49.57
Local financial unc.	5.82	3.95	58.93	55.97	37.58	19.05	**52.23**	57.06
Local economic unc.	6.02	4.13	48.81	45.80	33.67	22.01	36.94	42.21
CISS	5.93	4.13	48.81	44.40	45.57	25.35	40.76	45.31
No uncertainty	5.73	3.94	50.60	43.10	31.47	20.93	36.31	35.12
DAR(1)	**4.99**	**3.48**	55.36	56.81	20.53	16.83	33.12	40.33
RW	5.01	3.55	48.21	47.72	**18.89**	**14.47**	33.93	33.99

Bold figures indicate the best performance

## Conclusions

In this paper we obtain new indices measuring financial and economic uncertainty in the euro area, Germany, France, the UK and Austria, following the approach of Jurado et al. (2015), which measures uncertainty by the degree of predictability. We use monthly data comprising roughly 200 time series for the euro area and each country to construct our financial and economic uncertainty indices. The data cover the time span from January 2000 to December 2020.

After estimating the financial and economic uncertainty indices, we perform impulse response analyses in a vector error correction framework, where we focus on the impact of both local (country specific) and global (US) uncertainty shocks on industrial production, employment and the stock market, for the euro area, Germany, France, the UK and Austria. First, we observe significant negative effects of global financial uncertainty on industrial production, employment and the stock market, for the euro area and all countries considered. Second, for global economic uncertainty, we mostly observe a negative and statistically significant impact on the economic variables considered. Third, local financial uncertainty hardly shows statically significant effects on local industrial production, employment and the stock market. Forth, also for local economic uncertainty the effects are hardly significant. Only for the euro area, Germany and the UK local economic uncertainty shows a significant negative impact on the macroeconomic variables, however, only in the short run (Germany, UK).

In addition, we perform a forecasting analysis with respect to both loss-based and profit-based performance measures, where we assess the value added of our uncertainty indices in forecasting industrial production, employment and the stock market, for forecast horizons of one and twelve months. I.e., we compare the forecast performance of models including both local and global uncertainties, models including only local uncertainty and models including no uncertainty. We find that financial and/or economic uncertainty can improve the forecasting performance. Models including economic uncertainty improve the forecast performance for industrial production in the short run, while for the euro area, Germany and France models with financial uncertainty provide a value added for longer forecast horizons (twelve months). Regarding the forecasting of employment, models with economic uncertainty are among the best ones. Finally, a clear pattern can be observed when forecasting stock markets, considering profit-based performance measures. Models including both local and global financial uncertainties significantly outperform models including no uncertainty. In addition, models with both local and global financial uncertainties significantly outperform models with only local financial uncertainty, for a forecast horizon of one month, i.e., in the short-run global financial uncertainty seems to be more important than local uncertainty, when forecasting the stock market. Finally, for all countries but Germany, models with only local financial uncertainty significantly outperform models with no uncertainty, for a forecast horizon of twelve months.

## Appendix A: Financial and macroeconomic data

In the following, we provide details on the financial and macroeonomic data for the euro area and the corresponding transformations, which we use for computing the financial and economic uncertainty indices. Similar data and transformations are used for Germany, France, the United Kingdom and Austria. The data are available either at monthly frequencies or at daily frequencies, where daily are transformed to monthly frequencies by taking monthly averages. The employment data for the euro area and France are only available at a quarterly frequency. We construct monthly data from these quarterly series by estimating the missing values, following Shumway and Stoffer (1982) and Seong et al. (2013). A formal description is provided in the appendix of a prior version of this article, see Fortin et al. (2021). We consider 74 financial variables and 122 macroeconomic variables for the euro area. For Germany we use 74/120 financial/macroeconomic variables, for France 72/114, for the United Kingdom 76/114, and for Austria 77/122.

In order to ensure stationarity we perform various transformations. With respect to the financial data, we compute first differences (first diff) for interest rates, and spreads, i.e., differences (diff), for rates/yields. We calculate returns for stock/bond indices and foreign exchange rates in two ways: first we calculate returns of a month with respect to the previous month and annualize the results (monthly returns, m/m-1 (a)), second we calculate returns of a month with respect to the previous year (yearly returns, m/m-12). Finally we compute volatilities, namely stochastic volatilities (stoch vola), for the monthly returns of stock/bond indices and foreign exchange rates. We transform the macroeconomic data by taking yearly growth rates (m/m-12), the survey data are given in balances (difference between positive and negative answering options, measured as percentage points of total answers) and are not transformed.

The macroeconomic data include eight questions from the industry survey data collected by the DG ECFIN, for twelve different industries; hence, in total, 96 variables.^30^ The industries are beverages, wood (wood and wood and cork products except furniture, straw and plaiting materials), paper (paper and paper products), printing (printing and reproduction of recorded media), chemicals (chemicals and chemical products), rubber (rubber and plastics products), other minerals (other non-metallic mineral products), basic materials, fabricated metals (fabricated metal products except machinery and equipment), machinery (machinery and equipment N.E.C.), motor vehicles (motor vehicles, trailers and semi-trailers), and other manufacturing. The questions relate to the industrial confidence indicator, the production trend observed in recent months, order books, export order books, stocks of finished products, production expectations, selling price expectations, and employment expectations. The data used for calculating financial uncertainty indices are monthly and range from January 2000 to December 2020, i.e., 252 observations per variable.

Tables 3 and 4 list the financial and macroeconomic variables used for constructing the financial and economic uncertainty indices for the euro area. Similar data are used for Germany, France, the United Kingdom and Austria. Table 2 lists the abbreviations used in Tables 3 and 4.

**Table 2:** Abbreviations in tables with financial and macroeconomic data

Short	Name
BD	Bundesrepublik Deutschland, Germany
BIS	Bank for International Settlements
bo	Bond
BP	Basis points
CHF	Swiss franc
cor	Corporates
cur	Current prices
DG ECFIN	European Commission Directorate-General for Economic and Financial Affairs
DS	Datastream
EA	Euro area
ECB	European Central Bank
EBF	EBF/ACI FMA, EBF—European Banking Federation/ACI—The Financial Markets Association
Eur3	Euribor 3 m
exp orders	Export order books
fin	Financials
FX	Foreign exchange rate
GBP	British pound sterling
Gov	Government bond index
GovYie	Government bond yield
IBOXX	EURO IBOXX (euro area IBOXX bonds)
ind	Index
ind conf	Industrial confidence indicator
IRS	Interest rate swap
JPY	Japanes yen
m	Month, months
m/m-1 (a)	Monthly returns, annualized
m/m-12	Yearly returns
mio	Million
nsa	Not seasonally adjusted
orders	Order books
OIS	Overnight index swap
own	Own calculations
perc	Percent
prod trend	Production trend observed in recent months
rat	Ratio
Ref	Refinitiv
RI	Total return index
sa	Seasonally adjusted
Spr	Spread
stoch vol	Stochastic volatility of returns
thous	Thousand
Transform	Transformation
USD	US-dollar
vol	Volumes (in macroeconomic data)
vol	Volatility (in financial data)
w	Week, weeks
yie	Yield

**Table 3:** Financial data, euro area

	Name	Dimension	Transform	Source	Code
1	Eonia	Perc	First diff	EBF	EUEONIA
2	Euribor, 1 m	Perc	First diff	EBF	EIBOR1M
3	Euribor, 3 m	Perc	First diff	EBF	EIBOR3M
4	Euribor, 6 m	Perc	First diff	EBF	EIBOR6M
5	Euribor, 12 m	Perc	First diff	EBF	EIBOR1Y
6	Overnight index swap, 1w	Perc	First diff	Ref	OIEURSW
7	Overnight index swap, 2w	Perc	First diff	Ref	OIEUR2W
8	Overnight index swap, 3w	Perc	First diff	Ref	OIEUR3W
9	Overnight index swap, 1m	Perc	First diff	Ref	OIEUR1M
10	Overnight index swap, 2m	Perc	First diff	Ref	OIEUR2M
11	Overnight index swap, 3m	Perc	First diff	Ref	OIEUR3M
12	Overnight index swap, 4m	Perc	First diff	Ref	OIEUR4M
13	Overnight index swap, 5m	Perc	First diff	Ref	OIEUR5M
14	Overnight index swap, 6m	Perc	First diff	Ref	OIEUR6M
15	Overnight index swap, 7m	Perc	First diff	Ref	OIEUR7M
16	Overnight index swap, 8m	Perc	First diff	Ref	OIEUR8M
17	Overnight index swap, 9m	Perc	First diff	Ref	OIEUR9M
18	Overnight index swap, 10m	Perc	First diff	Ref	OIEUR10
19	Overnight index swap, 11m	Perc	First diff	Ref	OIEUR11
20	Overnight index swap, 12m	Perc	First diff	Ref	OIEUR1Y
21	Gov bond yield, EA, 5–7y	Perc	First diff	DS	AEMGVG3(RY)
22	Gov bond yield, EA, 7–10y	Perc	First diff	DS	AEMGVG4(RY)
23	Gov bond yield, EA, > 10y	Perc	First diff	DS	AEMGVG5(RY)
24	Gov bond yield, EA, 10y	Perc	First diff	ECB	EMGBOND
25	IBOXX Euro Fin	Perc	First diff	iBoxx	IBCFNAL(RY)
26	IBOXX Fin AAA	Perc	First diff	iBoxx	IBEFN3A(RY)
27	IBOXX Fin BBB	Perc	First diff	iBoxx	IBEFN3B(RY)
28	IBOXX Cor	Perc	First diff	iBoxx	IBCRPAL(RY)
29	IBOXX Cor AAA	Perc	First diff	iBoxx	IBC3AAL(RY)
30	IBOXX Cor BBB	Perc	First diff	iBoxx	IBC3BAL(RY)
31	IBOXX Non-Fin	Perc	First diff	iBoxx	IBCNFAL(RY)
32	IBOXX Non-Fin AAA	Perc	First diff	iBoxx	IBENF3A(RY)
33	IBOXX Non-Fin BBB	Perc	First diff	iBoxx	IBENF3B(RY)
34	IBOXX Sovereigns	Perc	First diff	iBoxx	IBSEUAL(RY)
35	Euro Stoxx index	Index	m/m-1 (a)	STOXX	DJEURST
36	Euro Stoxx dividend yield	Ratio	no	STOXX	DJEURST(DY)
37	Euro Stoxx price earn ratio	Ratio	no	STOXX	DJEURST(PE)
38	Euro Stoxx 50 index	Index	m/m-1 (a)	STOXX	DJES50I
39	Gov bond index, EA, 5–7y	RI	m/m-1 (a)	DS	AEMGVG3(RI)
40	Gov bond index, EA, 7–10y	RI	m/m-1 (a)	DS	AEMGVG4(RI)
41	Gov bond index, EA, g10y	RI	m/m-1 (a)	DS	AEMGVG5(RI)
42	USD/EUR	FX	m/m-1 (a)	ECB	USECBSP
43	JPY/EUR	FX	m/m-1 (a)	ECB	JPECBSP
44	CHF/EUR	FX	m/m-1 (a)	ECB	SWECBSP
45	GBP/EUR	FX	m/m-1 (a)	ECB	UKECBSP
46	Euro Stoxx index	Index	m/m-12	STOXX	DJEURST
47	Euro Stoxx 50 index	Index	m/m-12	STOXX	DJES50I
48	Gov bond index, EA, 5–7y	RI	m/m-12	DS	AEMGVG3(RI)
49	Gov bond index, EA, 7–10y	RI	m/m-12	DS	AEMGVG4(RI)
50	Gov bond index, EA, g10y	RI	m/m-12	DS	AEMGVG5(RI)
51	USD/EUR	FX	m/m-12	ECB	USECBSP
52	JPY/EUR	FX	m/m-12	ECB	JPECBSP
53	CHF/EUR	FX	m/m-12	ECB	SWECBSP
54	GBP/EUR	FX	m/m-12	ECB	UKECBSP
55	Spread GovYie, 10y, EA-BD	BP	Diff	ECB, DS	EMGBOND., BMBD10Y(RY)
56	Spread GovYie (EA, 10y)-Eur3	BP	Diff	ECB, EBF	EMGBOND., EIBOR3M
57	Spread GovYie, 10y, GR-BD	BP	Diff	DS	BMBD10Y(RY), BMBD10Y(RY)
58	Spread GovYie, 10y, IT-BD	BP	Diff	DS	BMIT10Y(RY) BMBD10Y(RY)
59	Libor-OIS-Spread, 1m	BP	Diff	EBF, Ref	EIBOR1M, OIEUR1M
60	Libor-OIS-Spread, 3m	BP	Diff	EBF, Ref	EIBOR3M, OIEUR3M
61	Libor-OIS-Spread, 6m	BP	Diff	EBF, Ref	EIBOR6M, OIEUR6M
62	Libor-OIS-Spread, 1y	BP	Diff	EBF, Ref	EIBOR1Y, OIEUR1Y
63	Spread fin: BBB-AAA	BP	Diff	iBoxx	IBEFN3B(RY), IBEFN3A(RY)
64	Spread cor: BBB-AAA	BP	Diff	iBoxx	IBC3BAL(RY), IBC3AAL(RY)
65	Spread non-fin: BBB-AAA	BP	diff	iBoxx	IBENF3B(RY), IBENF3A(RY)
66	Spread fin-sovereign	BP	Diff	iBoxx	IBCFNAL(RY), IBSEUAL(RY)
67	Euro Stoxx vola	Vola	Stoch vola	STOXX, own	DJEURST
68	Euro Stoxx 50 vola	Vola	Stoch vola	STOXX, own	DJES50I
69	Gov bond index, EA, 5–7y, vola	Vola	Stoch vola	DS, own	AEMGVG3(RI)
70	Gov bond index, EA, 7–10y, vola	Vola	Stoch vola	DS, own	AEMGVG4(RI)
71	USD/EUR vola	Vola	Stoch vola	ECB, own	USECBSP
72	JPY/EUR vola	Vola	Stoch vola	ECB, own	JPECBSP
73	CHF/EUR vola	Vola	Stoch vola	ECB, own	SWECBSP
74	GBP/EUR vola	Vola	Stoch vola	ECB, own	UKECBSP

When we compute the macroeconomic uncertainty indicator for the euro area the following financial variables are grouped with the macroeconomic variables, not with the financial variables: Euribor, 3 m; Euribor, 6 m; Euribor, 12 m; Government bond yield, EA, 5–7y; Government bond yield, EA, 7–10y; Government bond yield, EA, > 10y; Euro Stoxx index, m/m-1 (a); Euro Stoxx dividend yield; Euro Stoxx price earnings ratio; growth rates, m/m-1 (a), of USD/EUR, JPY/EUR, CHF/EUR, and GBP/EUR.

**Table 4:** Macroeconomic data, euro area

	Name	Dimension	Transform	Source	Code
1	Industrial confidence indicator	sa, balance	No	DG ECFIN	EKCNFBUSQ
2	Consumer confidence indicator	sa, balance	No	DG ECFIN	EKEUSCCIQ
3	Economic sentiment indicator	sa, index around 100	No	DG ECFIN	EKEUSESIG
4	Exports	sa, cur (mio euro)	m/m-12	Eurostat	EKEXPGDSB
5	Imports	sa, cur (mio euro)	m/m-12	Eurostat	EKIMPGDSB
6	Real effective exchange rate	index	m/m-12	ECB	EMXTW..RF
7	Money M3	sa, cur (mio euro)	m/m-12	ECB	EMM3....B
8	Bank loans for household consumption	nsa, cur (mio euro)	m/m-12	ECB	EMCRDCONA
9	Bank loans to non-fin corporations	nsa, cur (mio euro)	m/m-12	ECB	EMEBMC0.A
					EMEBMC1.A
					EMEBMC5.A
10	New car registrations	sa, volume (thous)	m/m-12	ECB	EKEBCARRO
11	Retail sales excl. motor veh & fuel	sa, turnover (index)	m/m-12	Eurostat	EKEW47MTG
12	Retail sales, nonfood prod excl. fuel	sa, turnover (index)	m/m-12	Eurostat	EKEW47PTG
13	Unemployed	sa, volume (thous)	m/m-12	Eurostat	EKESTUNPO
14	Unemployed, <25	sa, volume (thous)	m/m-12	Eurostat	Z8ES46XRO
15	Unemployed, females	sa, volume (thous)	m/m-12	Eurostat	Z8ESZAKBO
16	Unemployed, males	sa, volume (thous)	m/m-12	Eurostat	Z8ESQ6UQO
17	Unemployment rate	sa, percent	No	Eurostat	EKUN%TOTQ
18	Unemployment rate, <25	sa, percent	No	Eurostat	Z8ESZKEOQ
19	Unemployment rate, females	sa, percent	No	Eurostat	Z8ESJLSFQ
20	Unemployment rate, males	sa, percent	No	Eurostat	Z8ES29KYQ
21	Industrial production	sa, volume index	m/m-12	Eurostat	Z8ESQR59G
22	Industrial prod excl. construction	sa, volume index	m/m-12	Eurostat	EKIPTOT.G
23	Industrial prod manufacturing	sa, volume index	m/m-12	Eurostat	EKIPMAN.G
24	New order, manufacturing	sa, volume index	m/m-12	ECB	EKEBREMKG
25	New order, consumer durables	sa, volume index	m/m-12	ECB	EKEBREMHG
26	Harmonized index of cons prices	nsa, price index	m/m-12	Eurostat	EKCPHARMF
27	Producer price ind, mfc, dom mkts	nsa, price index	m/m-12	Eurostat	Z8ESR1E3F
28	Producer price ind, ind excl. constr	nsa, price index	m/m-12	Eurostat	EKPROPRCF
29	Industrial confidence, beverages	sa, balance	No	DG ECFIN	EK11.COBQ
30	Industrial confidence, wood	sa, balance	No	DG ECFIN	EK16.COBQ
31	Industrial confidence, paper	sa, balance	No	DG ECFIN	EK17.COBQ
32	Industrial confidence, printing	sa, balance	No	DG ECFIN	EK18.COBQ
33	Industrial confidence, chemicals	sa, balance	No	DG ECFIN	EK20.COBQ
34	Industrial confidence, rubber	sa, balance	No	DG ECFIN	EK22.COBQ
35	Industrial confidence, other minerals	sa, balance	No	DG ECFIN	EK23.COBQ
36	Industrial confidence, basic metals	sa, balance	No	DG ECFIN	EK24.COBQ
37	Industrial confidence, fabricated metals	sa, balance	No	DG ECFIN	EK25.COBQ
38	Industrial confidence, machinery	sa, balance	No	DG ECFIN	EK28.COBQ
39	Industrial confidence, motor vehicles	sa, balance	No	DG ECFIN	EK29.COBQ
40	Industrial confidence, other manufacturing	sa, balance	No	DG ECFIN	EK32.COBQ
41	Recent production trend, beverages	sa, balance	No	DG ECFIN	EK11.1.BQ
42	Recent production trend, wood	sa, balance	No	DG ECFIN	EK16.1.BQ
43	Recent production trend, paper	sa, balance	No	DG ECFIN	EK17.1.BQ
44	Recent production trend, printing	sa, balance	No	DG ECFIN	EK18.1.BQ
45	Recent production trend, chemicals	sa, balance	No	DG ECFIN	EK20.1.BQ
46	Recent production trend, rubber	sa, balance	No	DG ECFIN	EK22.1.BQ
47	Recent production trend, other minerals	sa, balance	No	DG ECFIN	EK23.1.BQ
48	Recent production trend, basic metals	sa, balance	No	DG ECFIN	EK24.1.BQ
49	Recent production trend, fabricated metals	sa, balance	No	DG ECFIN	EK25.1.BQ
50	Recent production trend, machinery	sa, balance	No	DG ECFIN	EK28.1.BQ
51	Recent production trend, motor vehicles	sa, balance	No	DG ECFIN	EK29.1.BQ
52	Recent production trend, other manufacturing	sa, balance	No	DG ECFIN	EK32.1.BQ
53	Order books, beverages	sa, balance	No	DG ECFIN	EK11.2.BQ
54	Order books, wood	sa, balance	No	DG ECFIN	EK16.2.BQ
55	Order books, paper	sa, balance	No	DG ECFIN	EK17.2.BQ
56	Order books, printing	sa, balance	No	DG ECFIN	EK18.2.BQ
57	Order books, chemicals	sa, balance	No	DG ECFIN	EK20.2.BQ
58	Order books, rubber	sa, balance	No	DG ECFIN	EK22.2.BQ
59	Order books, other minerals	sa, balance	No	DG ECFIN	EK23.2.BQ
60	Order books, basic metals	sa, balance	No	DG ECFIN	EK24.2.BQ
61	Order books, fabricated metals	sa, balance	No	DG ECFIN	EK25.2.BQ
62	Order books, machinery	sa, balance	No	DG ECFIN	EK28.2.BQ
63	Order books, motor vehicles	sa, balance	No	DG ECFIN	EK29.2.BQ
64	Order books, other manufacturing	sa, balance	No	DG ECFIN	EK32.2.BQ
65	Export order books, beverages	sa, balance	No	DG ECFIN	EK11.3.BQ
66	Export order books, wood	sa, balance	No	DG ECFIN	EK16.3.BQ
67	Export order books, paper	sa, balance	No	DG ECFIN	EK17.3.BQ
68	Export order books, printing	sa, balance	No	DG ECFIN	EK18.3.BQ
69	Export order books, chemicals	sa, balance	No	DG ECFIN	EK20.3.BQ
70	Export order books, rubber	sa, balance	No	DG ECFIN	EK22.3.BQ
71	Export order books, other minerals	sa, balance	No	DG ECFIN	EK23.3.BQ
72	Export order books, basic metals	sa, balance	No	DG ECFIN	EK24.3.BQ
73	Export order books, fabricated metals	sa, balance	No	DG ECFIN	EK25.3.BQ
74	Export order books, machinery	sa, balance	No	DG ECFIN	EK28.3.BQ
75	Export order books, motor vehicles	sa, balance	No	DG ECFIN	EK29.3.BQ
76	Export order books, other manufacturing	sa, balance	No	DG ECFIN	EK32.3.BQ
77	Stocks of finished products, beverages	sa, balance	No	DG ECFIN	EK11.4.BQ
78	Stocks of finished products, wood	sa, balance	No	DG ECFIN	EK16.4.BQ
79	Stocks of finished products, paper	sa, balance	No	DG ECFIN	EK17.4.BQ
80	Stocks of finished products, printing	sa, balance	No	DG ECFIN	EK18.4.BQ
81	Stocks of finished products, chemicals	sa, balance	No	DG ECFIN	EK20.4.BQ
82	Stocks of finished products, rubber	sa, balance	No	DG ECFIN	EK22.4.BQ
83	Stocks of finished products, other minerals	sa, balance	No	DG ECFIN	EK23.4.BQ
84	Stocks of finished products, basic metals	sa, balance	No	DG ECFIN	EK24.4.BQ
85	Stocks of finished products, fabricated metals	sa, balance	No	DG ECFIN	EK25.4.BQ
86	Stocks of finished products, machinery	sa, balance	No	DG ECFIN	EK28.4.BQ
87	Stocks of finished products, motor vehicles	sa, balance	No	DG ECFIN	EK29.4.BQ
88	Stocks of finished products, other manufacturing	sa, balance	No	DG ECFIN	EK32.4.BQ
89	Production expectations, beverages	sa, balance	No	DG ECFIN	EK11.5.BQ
90	Production expectations, wood	sa, balance	No	DG ECFIN	EK16.5.BQ
91	Production expectations, paper	sa, balance	No	DG ECFIN	EK17.5.BQ
92	Production expectations, printing	sa, balance	No	DG ECFIN	EK18.5.BQ
93	Production expectations, chemicals	sa, balance	No	DG ECFIN	EK20.5.BQ
94	Production expectations, rubber	sa, balance	No	DG ECFIN	EK22.5.BQ
95	Production expectations, other minerals	sa, balance	No	DG ECFIN	EK23.5.BQ
96	Production expectations, basic metals	sa, balance	No	DG ECFIN	EK24.5.BQ
97	Production expectations, fabricated metals	sa, balance	No	DG ECFIN	EK25.5.BQ
98	Production expectations, machinery	sa, balance	No	DG ECFIN	EK28.5.BQ
99	Production expectations, motor vehicles	sa, balance	No	DG ECFIN	EK29.5.BQ
100	Production expectations, other manufacturing	sa, balance	No	DG ECFIN	EK32.5.BQ
101	Selling price expectations, beverages	sa, balance	No	DG ECFIN	EK11.6.BQ
102	Selling price expectations, wood	sa, balance	No	DG ECFIN	EK16.6.BQ
103	Selling price expectations, paper	sa, balance	No	DG ECFIN	EK17.6.BQ
104	Selling price expectations, printing	sa, balance	No	DG ECFIN	EK18.6.BQ
105	Selling price expectations, chemicals	sa, balance	No	DG ECFIN	EK20.6.BQ
106	Selling price expectations, rubber	sa, balance	No	DG ECFIN	EK22.6.BQ
107	Selling price expectations, other minerals	sa, balance	No	DG ECFIN	EK23.6.BQ
108	Selling price expectations, basic metals	sa, balance	No	DG ECFIN	EK24.6.BQ
109	Selling price expectations, fabricated metals	sa, balance	No	DG ECFIN	EK25.6.BQ
110	Selling price expectations, machinery	sa, balance	No	DG ECFIN	EK28.6.BQ
111	Selling price expectations, motor vehicles	sa, balance	No	DG ECFIN	EK29.6.BQ
112	Selling price expectations, other manufacturing	sa, balance	No	DG ECFIN	EK32.6.BQ
113	Employment expectations, paper	sa, balance	No	DG ECFIN	EK17.7.BQ
114	Employment expectations, printing	sa, balance	No	DG ECFIN	EK18.7.BQ
115	Employment expectations, chemicals	sa, balance	No	DG ECFIN	EK20.7.BQ
116	Employment expectations, rubber	sa, balance	No	DG ECFIN	EK22.7.BQ
117	Employment expectations, other minerals	sa, balance	No	DG ECFIN	EK23.7.BQ
118	Employment expectations, basic metals	sa, balance	No	DG ECFIN	EK24.7.BQ
119	Employment expectations, fabricated metals	sa, balance	No	DG ECFIN	EK25.7.BQ
120	Employment expectations, machinery	sa, balance	No	DG ECFIN	EK28.7.BQ
121	Employment expectations, motor vehicles	sa, balance	No	DG ECFIN	EK29.7.BQ
122	Employment expectations, other manufacturing	sa, balance	No	DG ECFIN	EK32.7.BQ

The variable bank loans to non-financial corporations is created by summing the three variables bank loans to non-financial corporations < 1 year (EMEBMC0.A), bank loans to non-financial corporations 1–4 years (EMEBMC1.A), and bank loans to non-financial corporations > 4 years (EMEBMC5.A), and then computing growth rates. Two variables of the survey data relating to employment expectations are not available for the euro area because the data only start later than January 2000. These are the series related to beverages (EK11.7.BQ) and wood (EK16.7.BQ).

## Appendix B: Empirical results

### Impulse response analysis

In the following, we present graphs showing the impulse response functions for Germany, France, the United Kingdom and Austria. We present the responses of local industrial production, employment and the stock market to one standard deviation shocks in (i) the local and global financial uncertainty indices, and (ii) the local and global economic uncertainty indices (Figs. 6, 7, 8, 9).

### Forecasting analysis

In the following, we present forecast performance measures for Germany, France, the UK and Austria, when forecasting industrial production, employment and the stock market (Tables 5, 6, 7 and 8).

**Table 5:** Forecasting industrial production, employment and the stock market in Germany

	RMSE	MAE	DA	DV	RMSE	MAE	DA	DV
	$h=1$				$h=12$			
	*Industrial production, DE*							
Local & global financial unc	2.76	1.51	**58**.**33**	58.60	10.95	7.61	54.78	**44**.**29**
Local & global economic unc	**2**.**15**	**1**.**38**	55.95	**68**.**12**	10.27	6.58	54.14	39.91
Local financial unc	3.09	1.56	54.17	55.05	9.53	6.99	53.50	38.81
Local economic unc	2.46	1.45	55.36	62.91	9.68	6.26	43.31	24.29
CISS	2.94	1.51	57.14	60.63	11.79	7.09	**55**.**41**	41.06
No uncertainty	3.04	1.54	53.57	50.67	8.70	5.50	52.87	39.75
DAR(1)	3.10	1.51	50.00	42.25	7.93	5.06	52.23	38.61
RW	2.46	1.41	45.83	47.17	**7**.**33**	**4**.**85**	51.19	42.52
	*Employment, DE*							
Local & global financial unc	0.10	0.06	85.71	89.05	0.83	0.61	82.80	84.06
Local & global economic unc	**0**.**09**	0.06	**89**.**29**	**94**.**09**	0.92	0.60	88.54	92.21
Local financial unc	0.11	0.07	85.12	84.69	0.70	0.48	89.81	92.63
Local economic unc	0.09	0.06	86.31	91.09	0.71	0.49	87.26	90.32
CISS	0.10	0.06	86.31	87.48	0.71	0.48	89.81	92.24
No uncertainty	0.10	0.06	85.71	91.16	**0**.**62**	**0**.**42**	**92**.**36**	**93**.**59**
DAR(1)	0.10	**0**.**06**	**89**.**29**	89.68	0.71	0.49	87.90	92.23
RW	0.12	0.10	86.90	85.05	1.05	0.99	79.17	83.97
	*DAX 30*							
Local & global financial unc	6.02	4.03	**66**.**07**	**68**.**72**	48.63	25.91	61.15	**65**.**60**
Local & global economic unc	6.81	4.55	55.36	55.68	45.57	21.86	54.14	49.24
Local financial unc	6.03	4.06	56.55	52.68	28.97	17.69	**64**.**97**	62.00
Local economic unc	6.17	4.29	56.55	54.73	24.18	18.22	56.05	54.48
CISS	5.97	3.98	62.50	60.32	33.11	21.27	55.41	52.49
No uncertainty	5.76	3.90	57.14	54.62	23.11	16.79	63.69	60.61
DAR(1)	**5**.**21**	**3**.**65**	60.12	56.96	19.47	15.84	62.42	61.97
RW	5.22	3.76	63.10	55.41	**18**.**90**	**15**.**60**	49.40	49.78

Bold figures indicate the best performance

**Table 6:** Forecasting industrial production, employment and the stock market in France

	RMSE	MAE	DA	DV	RMSE	MAE	DA	DV
	$h=1$				$h=12$			
	*Industrial production, FR*							
Local & global financial unc	6.73	2.01	61.31	58.21	7.65	4.43	**57**.**32**	**62**.**94**
Local & global economic unc	5.56	1.98	61.90	**67**.**16**	8.00	4.62	42.04	33.27
Local financial unc	8.12	2.20	64.29	60.58	7.95	4.39	50.32	40.06
Local economic unc	8.02	2.34	61.31	58.90	7.93	4.48	45.86	29.67
CISS	7.41	2.09	**66**.**67**	60.99	12.77	5.93	50.32	44.12
No uncertainty	8.16	2.31	66.07	59.97	8.15	4.57	47.13	32.12
DAR(1)	6.13	1.91	64.29	59.59	7.24	3.95	41.40	22.05
RW	**4**.**16**	**1**.**70**	50.00	40.07	**6**.**98**	**3**.**57**	42.26	29.80
	*Employment, FR*							
Local & global financial unc	0.17	0.06	88.10	86.93	0.89	0.63	81.53	80.77
Local & global economic unc	**0**.**13**	**0**.**05**	91.07	97.13	0.79	0.54	**87**.**26**	85.65
Local financial unc	0.17	0.06	89.88	94.41	0.85	0.58	86.62	85.94
Local economic unc	0.17	0.06	91.07	94.55	**0**.**76**	**0**.**52**	85.99	85.20
CISS	0.16	0.05	89.88	94.25	1.13	0.64	**87**.**26**	85.81
No uncertainty	0.17	0.05	90.48	87.30	0.78	0.54	85.99	84.01
DAR(1)	0.16	0.05	**94**.**64**	**97**.**29**	0.77	0.53	83.44	82.24
RW	0.17	0.09	81.55	71.03	0.83	0.70	82.74	**86**.**28**
	*CAC 40*							
Local & global financial unc	6.16	4.06	**60**.**12**	**65**.**61**	28.11	20.37	55.41	64.94
Local & global economic unc	7.35	4.48	52.38	49.29	31.26	18.89	49.68	54.11
Local financial unc	6.83	4.25	54.17	50.07	39.47	19.23	**64**.**33**	**71**.**72**
Local economic unc	6.67	4.06	52.98	53.50	22.70	17.80	40.13	47.84
CISS	6.53	4.25	52.38	59.31	56.12	25.56	37.58	37.05
No uncertainty	6.27	4.07	52.38	52.11	25.52	18.77	38.22	38.13
DAR(1)	**4**.**90**	**3**.**43**	55.36	53.41	20.00	16.46	39.49	37.02
RW	4.93	3.50	49.40	46.23	**18**.**45**	**14**.**50**	41.67	37.74

Bold figures indicate the best performance

**Table 7:** Forecasting industrial production, employment and the stock market in the UK

	RMSE	MAE	DA	DV	RMSE	MAE	DA	DV
	$h=1$				$h=12$			
	*Industrial production, UK*							
Local & global financial unc	2.63	1.30	45.83	48.82	5.12	3.85	46.50	43.06
Local & global economic unc	2.29	1.26	50.00	**56**.**50**	5.93	3.88	57.96	51.52
Local financial unc	2.60	1.26	50.60	53.26	5.69	4.08	44.59	42.19
Local economic unc	**2**.**20**	1.25	45.24	55.31	6.04	4.26	50.96	49.04
CISS	2.44	1.27	48.21	52.12	5.62	4.09	**61**.**15**	**60**.**10**
No uncertainty	2.36	1.24	45.83	50.65	5.36	3.97	47.77	44.25
DAR(1)	3.04	1.25	**55**.**95**	48.98	4.57	3.51	44.59	45.25
RW	2.24	**1**.**12**	50.60	44.27	**4**.**20**	**3**.**17**	45.83	50.88
	*Employment, UK*							
Local & global financial unc	0.20	0.15	71.43	75.63	1.25	0.92	79.62	81.98
Local & global economic unc	0.19	0.14	**75**.**60**	**81**.**37**	**1**.**06**	**0**.**80**	**83**.**44**	87.21
Local financial unc	0.20	0.15	72.02	75.21	1.40	0.98	81.53	84.01
Local economic unc	0.18	0.14	73.21	78.80	1.56	1.09	76.43	78.09
CISS	0.21	0.15	67.86	70.75	1.43	1.08	77.07	78.41
No uncertainty	0.20	0.14	69.05	73.87	1.35	1.04	77.71	79.67
DAR(1)	**0**.**18**	**0**.**14**	75.00	77.18	1.32	0.99	78.98	80.76
RW	0.20	0.16	67.26	67.54	1.39	1.25	79.76	**87**.**30**
	*FTSE 100*							
Local & global financial unc	4.48	3.20	**60**.**71**	**67**.**46**	32.03	17.94	55.41	**56**.**56**
Local & global economic unc	5.24	3.62	49.40	46.86	28.75	15.46	52.87	37.31
Local financial unc	4.89	3.22	52.98	51.75	41.48	18.10	**56**.**69**	47.12
Local economic unc	4.73	3.26	48.81	48.97	23.59	14.43	43.95	31.92
CISS	4.54	3.10	47.02	41.19	28.06	15.39	43.95	38.47
No uncertainty	4.59	3.14	48.81	49.06	22.42	13.69	49.68	38.90
DAR(1)	4.13	2.85	51.79	49.72	15.30	11.31	54.14	46.76
RW	**4**.**09**	**2**.**80**	53.57	46.79	**14**.**66**	**11**.**09**	38.69	37.95

Bold figures indicate the best performance

**Table 8:** Forecasting industrial production, employment and the stock market in Austria

	RMSE	MAE	DA	DV	RMSE	MAE	DA	DV
	$h=1$				$h=12$			
	*Industrial production, AT*							
Local & global financial unc	2.27	1.51	47.62	53.50	6.57	4.95	61.15	57.05
Local & global economic unc	**2**.**10**	1.48	52.98	**54**.**99**	7.69	5.49	63.69	60.39
Local financial unc	2.32	1.51	47.62	49.44	7.09	5.15	59.24	51.29
Local economic unc	2.38	1.52	52.38	46.66	8.01	5.10	52.23	43.85
CISS	2.42	1.51	50.60	51.32	13.38	6.09	65.61	57.99
No uncertainty	2.40	1.47	50.60	49.86	6.53	4.36	64.97	56.88
DAR(1)	2.56	1.39	**57**.**74**	49.18	5.96	**3**.**89**	68.15	61.24
RW	2.21	**1**.**34**	50.60	45.35	**5**.**38**	3.92	**69**.**64**	**72**.**81**
	*Employment, AT*							
Local & global financial unc	0.58	0.29	62.50	57.49	1.70	1.22	76.43	71.49
Local & global economic unc	0.64	0.30	63.69	56.63	1.71	1.25	84.71	80.28
Local financial unc	0.62	0.30	57.74	49.03	1.65	1.18	80.89	76.27
Local economic unc	0.65	0.30	60.71	**68**.**82**	1.67	1.11	81.53	76.45
CISS	0.60	0.28	58.93	52.76	1.63	1.09	83.44	78.12
No uncertainty	0.59	0.28	61.31	54.60	1.53	1.02	84.08	79.32
DAR(1)	0.58	**0**.**27**	59.52	54.09	**1**.**51**	**1**.**02**	84.71	78.83
RW	**0**.**55**	0.28	**64**.**88**	61.77	1.63	1.42	**85**.**12**	**90**.**87**
	*ATX*							
Local & global financial unc	**5**.**95**	4.43	**65**.**48**	**71**.**42**	65.13	30.57	57.96	55.04
Local & global economic unc	7.24	5.13	51.79	56.26	90.81	37.47	46.50	42.79
Local financial unc	6.89	4.87	57.14	60.60	58.95	27.84	**62**.**42**	**56**.**19**
Local economic unc	7.28	4.93	51.19	55.66	43.78	29.89	40.76	32.72
CISS	6.92	4.84	55.95	62.27	117.63	42.66	45.22	42.67
No uncertainty	6.78	4.65	55.36	61.38	41.61	28.39	44.59	33.78
DAR(1)	6.40	**4**.**18**	58.93	58.84	35.02	26.08	35.03	23.56
RW	6.61	4.40	47.62	38.54	**28**.**59**	**21**.**02**	41.67	38.86

Bold figures indicate the best performance

## Footnotes

1 The ECB’s indicators use different weighting schemes to aggregate individual variables or subindices into one index: weights reflecting the time-varying cross-correlation structure (CISS) or equal weights (new CISS), see Holló et al. (2012). The CISS is computed for the euro area as a whole on a weekly basis, the new CISS is computed for the euro area as a whole and for all euro area countries on a daily basis.

2 The proper measurement of uncertainty requires removing the forecastable component ${{\mathbb {E}}}\left( y_{j,t+h} | I_t \right)$ *before* computing conditional volatility. Otherwise the forecastable variation would be (falsely) classified as uncertain.

3 To simplify notation we use ${{\mathbb {E}}}_t (\cdot)$ to denote ${{\mathbb {E}}}[\cdot | I_t]$.

4 Following JLN, we choose polynomials of order four for $\Phi _j^y(L)$ and polynomials of order two for $\gamma _j^F(L)$ and $\gamma _j^W(L)$.

5 We choose the factor in $x_{it}^2$ corresponding to the largest eigenvalue.

6 To estimate stochastic volatility in the forecast errors we use the ‘stochvol’ R package (see Kastner 2016).

7 Following JLN, we choose an order of four in the autoregressive dynamics of the factors to coincide with the order of polynomial $\Phi _j^y(L)$, i.e., $q=4$.

8 In total, the survey data cover seven questions relating to (i) production trend observed in recent months, (ii) order books, (iii) export order books, (iv) stocks of finished products, (v) production expectations, (vi) selling price expectations, (vii) employment expectations, and one overall variable, the industrial confidence indicator.

9 For more details on real-time macroeconomic data see, for example, Croushore and Stark (2000).

10 Thus, $N^x=196$ for the euro area, $N^x=194$ for Germany, $N^x=186$ for France, $N^x=190$ for the UK, and $N^x=199$ for Austria.

11 Note that the forecast tends to the unconditional mean as the forecast horizon tends to infinity.

12 Less diversity among the financial uncertainty indices is mainly due to the fact that a lot of financial variables are identical over all countries except the UK, as they are related to the euro area, e.g., money market interest rates and exchange rates.

13 The descriptive statistics for the euro area and all countries under consideration can be obtained upon request.

14 To simplify notation we drop the country index from Eq. (11).

15 In 2021 the DAX 30 was redesigned to include 40 stocks and is now called DAX 40.

16 We use total financial uncertainty and total macro uncertainty, see https://www.sydneyludvigson.com/macro-and-financial-uncertainty-indexes.

17 In most cases the lag length was one, i.e., ${\hat{p}}=1$. Hence we proceeded with a lag of one for all models, also due to the curse of dimensionality.

18 The first vector has one as the first component and zeros elsewhere and the second vector has one as the second component and zeros elsewhere.

19 However, with larger models some of the results seem to become unstable. This would also be the case if we included all financial and economic (global and local) uncertainty indices in one large model.

20 For the impulse response analysis and the forecasting analysis we use EViews 13.

21 We thank an anonymous referee for the idea to include a global uncertainty indicator to get a better picture of the influence of global and/or local uncertainty.

22 As all forecasted variables are integrated of order one, we apply the AR(1) model on log differences of the underlying variable.

23 Note that in case when only one uncertainty index is included, or the CISS (see cases (iii)–(v)) the number of stationary variables in (11) reduces to one and number of cointegrating vectors reduces to two, i.e., ${\hat{r}}=2$, where the first cointegrating vector is the first canonical basis vector.

24 In this case all variables in (11) are integrated of order one and ${\hat{r}}=1$.

25 The model with the lowest RMSE over the total period, the random walk, performs much worse than any of the three models during the global financial crisis.

26 The only exception for the UK is the largest DV for a forecast horizon of twelve months, which is achieved by the random walk model.

27 Thus, whether including global uncertainty improves the forecast performance.

28 For Germany this applies only for a forecast horizon of one month.

29 For Austria this is also true when considering the RMSE, for a one-month forecast horizon. This is the only case when we find a significant result for a loss measure.
